# Heparinization of anodized aluminum oxide (AAO) membranes for affinity separations of biocolloids

**DOI:** 10.1039/d6ra07687d

**Published:** 2026-10-20

**Authors:** Kayla E. Daugherty, Logan D. Martin, Mara Leach-Baughman, Kearstin L. Edmonds, Rebecca E. Dutch, Malgorzata Chwatko, Stephen E. Rankin, Barbara L. Knutson

**Affiliations:** a University of Kentucky, Department of Chemical & Materials Engineering Lexington KY 40406-0046 USA stephen.rankin@uky.edu bknut2@uky.edu; b University of Kentucky, Department of Molecular and Cellular Biochemistry Lexington KY 40506 USA

## Abstract

Affinity membranes are developed by the immobilization of heparin onto anodized aluminum oxide (AAO) supports *via* amine grafting followed by amide coupling. Optimized procedures give heparinized AAO (HEP–AAO) membranes with a stable layer of 0.832–1.33 mg heparin per m^2^ pore area in 224 nm diameter pores. Following the heparinization of the membrane, the bioavailability of heparin is demonstrated through the capture and release of fibroblast growth factor 2 (FGF2), a protein for which heparin acts as a ligand. The capping of unreacted amines using citraconic anhydride reduces nonspecific protein binding without loss of heparin activity. The pore accessibility of colloidal particles is confirmed through the electrostatically-driven binding and release of 20 nm fluorescent amine-grafted silica nanoparticles, which are used as a model system to investigate the capture and release of biocolloids. Application of heparinized AAO membranes for the affinity capture and release of adeno associated virus 2 (AAV-2) is demonstrated in tangential flow filtration. Preliminary results for the affinity capture of AAV-2 pre-filtered culture medium capture significant AAV2 with negligible retention of host protein and DNA, paving a path for further optimization of viral vector purification.

## Abstract 2

Heparinized anodized aluminum oxide membranes are investigated for the purification of AAV2 viral vectors through a series of affinity capture, rinse and release steps.

## Graphical abstract

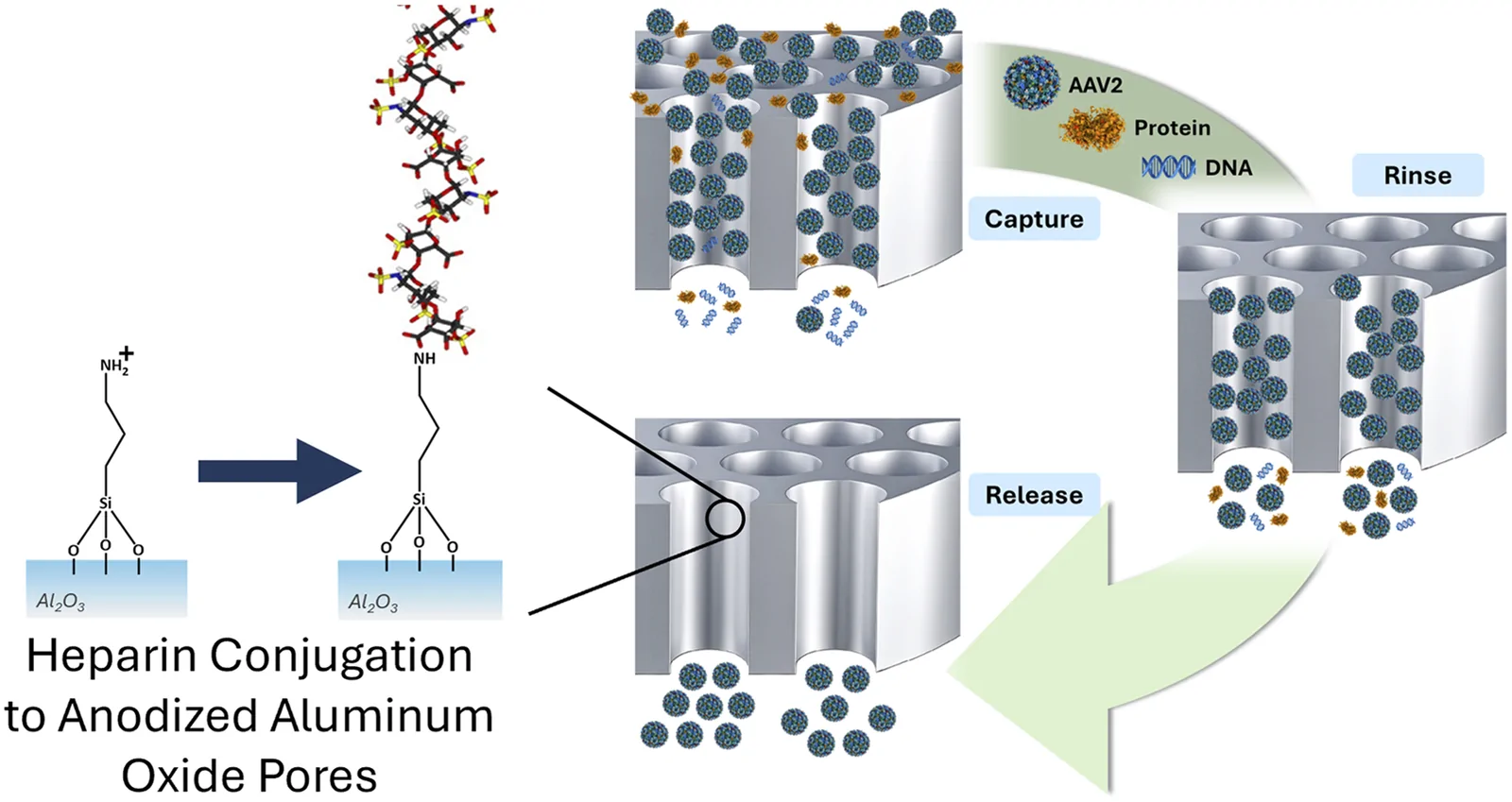

## Introduction

Biologics, such as viral vectors, have seen increased therapeutic application in gene therapies and gene treatment.^1,2^ This growth in demand has highlighted the labor- and time-intensive manufacturing process for such therapeutics, translating to prohibitive costs for patients, and thus necessitating the development of more efficient and cost-effective methods. Examples of treatments that were recently approved by the FDA are Luxturna, Zolgensma, Tecartus, and Hemgenix, costing anywhere from several hundred thousand to several millions of dollars per treatment.^3–5^ Over 60% of the total costs for biopharmaceutical productions can be attributed to downstream purification processes.^6^ Specifically, the downstream purification of viral vectors accounts for over 70% of their total cost.^7^

As a strategy to reduce cost and complexity of purification of biologics, affinity separations utilize the reversible molecular interactions between proteins and ligands, allowing the targeted separation of molecules and biologics in mixtures. Separations of biologics traditionally employ ultracentrifugation and packed-bed or gel column chromatography.^8^ However, drawbacks with current approaches include high pressure drop, long processing times, and low affinity ligand utilization due to diffusion-limited mass transport.^9^ One strategy to improve downstream purification is replacing affinity column chromatography with affinity membrane chromatography. The use of affinity membrane columns can maintain the bioactivity of biologics by decreasing back pressure through a low bed height.^7^ Affinity membrane chromatography accelerates processing by providing high throughput, scalability, and increased ligand use from convective flow through their pores, allowing for a higher dynamic binding capacity.^6,7^ A techno-economic analysis simulation conducted by Romero *et al.* reported that increasing the capacity of a column through increased accessible surface area could substantially reduce the cost associated with processing.^10^

While cellulose-based membranes are most commonly used in affinity membrane chromatography, inorganic membranes are advantageous for bioprocessing because of their stability over a wide range of chemical, thermal, and mechanical conditions.^9,11^ Anodized aluminum oxide (AAO) membranes are specifically desirable because of their homogeneous morphology, high pore density, and well-ordered, non-tortuous pore structure.^12^ The hydroxyl groups present on the surface make them ideal for further functionalization, enabling tunability for affinity-based application.^13^ While AAO systems suffer from relative stiffness and fragility relative to polymeric membranes, advances in the synthesis of mesh patterned AAO and tubular AAO modules have made them viable technologies for bioseparations.^14–16^ Shi *et al.* demonstrated the capture of a model protein, hemoglobin, using chitosan-functionalized AAO membranes.^17^ While functionalized AAO has previously been demonstrated for affinity-based separations of proteins, it is relatively unexplored for affinity capture of larger biocolloids, such as viral vectors.

Inorganic virus-sized particles allow for the investigation of particle capture and release for membrane development without the use of viral vectors. Virus-like particles (VLPs) are traditionally protein-based nanoparticles, which are tunable through the selection of proteins and other biomaterials used to synthesize them.^18,19^ However, biotic VLPs require labor and time intensive production, purification, and storage processes, similar to the viral vectors themselves.^20,21^ Virus-sized silica nanoparticles (SNPs) are applicable as inorganic model VLPs. The facile synthesis process of SNPs allow for the adjustment of morphology and size, and their surface chemistry is readily tunable.^22^ The addition of amines to the surface provides increased biocompatibility and enables the attachment of other compounds, such as proteins, ligands, and other biologics, as well as fluorescent dyes.^23^ While virus-sized SNPs do not mimic the specific biological interactions and mechanical behavior of VLPs and viral vectors, their size and surface composition are highly reproducible and allow for the demonstration of particle size effects, surface functionalization and charge on surface capture and release.^24^

The affinity ligand investigated in this study, heparin, is a bioactive glycosaminoglycan ligand containing negative sulfate groups.^25^*In vivo*, it binds to proteins to regulate biological activities.^26^ Song *et al.* previously developed an affinity membrane for blood purification by attaching heparin to a nylon membrane.^27^ Heparin has an affinity for the adeno-associated virus (AAV), and specifically AAV2, a commonly used viral vector^28^ approximately 20 nm in diameter. Heparin affinity resins have been previously used for viral vector purification. Wang *et al.* also used heparinized cellulose membranes to observe the bioactivity of isolated AAV2, but they did not quantify separation performance.^29^

Here we report the synthesis of heparin-functionalized AAO membranes and their testing for electrostatic and affinity-based separations, leading towards affinity-driven purification of viral vectors with AAV2 as a model. Heparin is covalently attached to AAO *via* an amine coupling agent. Dye-based electrostatic assays quantify the surface density of accessible amines and heparin, where heparinization imparts a more negative charge on the surface relative to the positively charged amines. The bioactivity of immobilized heparin is demonstrated through affinity-driven binding of a heparin-selective protein, Fibroblast Growth Factor 2 (FGF2). The electrostatically-driven capture and release of 20 nm SNPs in AAO (224 nm pores) is used to explore the capacity and the recovery of model biocolloids on heparinized AAO (HEP–AAO) membranes. In a preliminary investigation, the affinity-based recovery of AAV2 from clarified cell lysate containing host DNA and protein using HEP–AAO is examined as part of a lab-scale membrane-based separation train (Fig. 1).

**Fig. 1:**
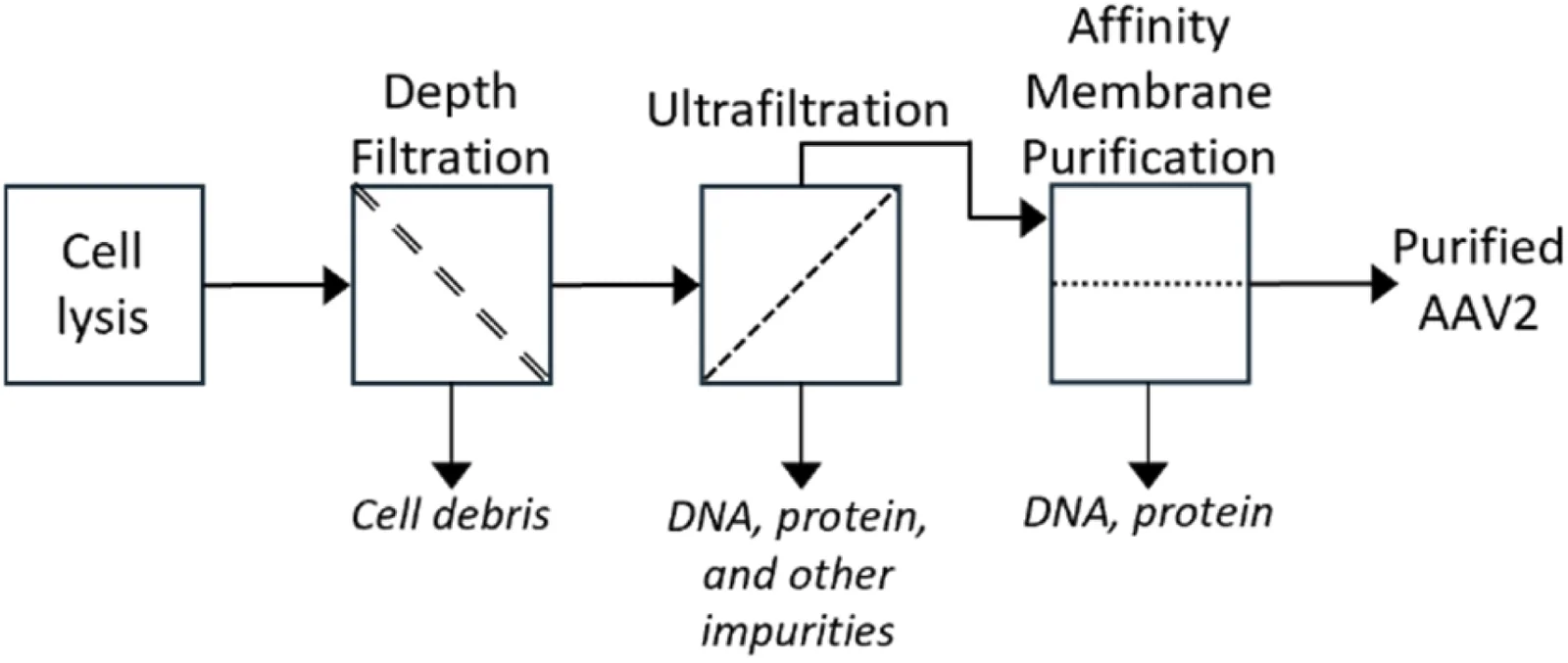
Process model of lab-scale AAV2 separation train. Prior to the affinity membrane, AAV2 cell lysate was processed by depth filtration and ultrafiltration to remove impurities such as cell debris, DNA, protein, and other biologics. Affinity membrane purification produced a purified AAV2 product.

## Experimental

### Materials and methods

#### Materials

The AAO membranes used here were Whatman Anodisc 25 mm membranes with a nominal 0.2 µm pore diameter (Cytiva). AAO membranes were functionalized using the following chemicals and solvents: 3-triethoxysilyl-propylamine (APTES, Sigma-Aldrich), hydrogen peroxide (30% GR ACS, Supelco), heparin sodium salt from porcine intestinal mucosa (IU > 100/mg and MW of 16.4 kDa, Thermo Scientific), *N*-(3-dimethylaminopropyl)-*N*′-ethylcarbodiimide (EDC, 98%, Thermo Scientific Chemicals), *N*-hydroxysuccinimide (NHS, 98+% Thermo Scientific Chemicals), 2-(*N*-morpholino)ethanesulfonic acid monohydrate (MES, Ultrapure, Thermo Scientific), and citraconic anhydride 98% (Thermo Scientific Chemicals). Silica nanoparticles (SNP) were synthesized and functionalized using tetraethyl orthosilicate (TEOS, 99.9% Thermo Scientific), anhydrous ethanol (Koptec), ammonium hydroxide (ACS GR, VWR BDH Chemicals), methanol (ACS GR, VWR BDH Chemicals), nitric acid (Supelco) and fluorescein isothiocyanate, isomer 1 (FITC, 95%, Thermo Scientific). Ultrafiltered (UF) water was produced from a MilliporeSigma Milli-Q Advantage A10 purification system and Biopak polisher. Controlled pH solutions were synthesized by adjusting UF water with ammonium hydroxide or hydrochloric acid ACS GR (VWR BDH Chemicals).

Functionalized AAO membranes and SNPs were characterized using acid orange II (AO II, Thermo Scientific) and methylene blue (MB, Aldrich Chemical Company). The bioactivity of immobilized heparin was demonstrated using human FGF2 basic protein (FGF2, premium grade, Acro Biosystems) and FGF2 polyclonal antibody, FITC conjugated (Ab-FITC, Bioss Antibodies) in aqueous solutions of Tris–HCl (Biotechnology VWR) and sodium chloride (Fisher Chemical). A cell culture suspension of AAV2 for purification experiments, grown in T175 flasks and resuspended in Dulbecco's Modified Eagle Medium (DMEM, Thermo Fisher Scientific), was purchased from the University of North Carolina (UNC) Vector Core.^30^

#### Methods

##### AAO characterization

Membranes were imaged using a FEI Helios Nanolab 660. ImageJ was used to measure pore diameters. Porosity was determined from pressure-driven flux measurements of UF water using a Sterlitech HP4750 stainless steel dead-end filtration cell, with feed pressure (30–60 kPa) provided by ultrahigh purity nitrogen.

##### AAO functionalization and quantification

AAO membranes were cleaned and hydroxylated prior to amine functionalization using a modification of the procedure of Jani *et al.*^31^ Specifically, the AAO was submersed in boiling hydrogen peroxide for 30 min, rinsed with UF water, and dried overnight at 60 °C.

Amines were covalently attached to hydroxylated AAO by submersion in a 100 mL solution of APTES in toluene (1.5% v/v) for 2 h with moderate agitation, followed by placement under vacuum for 30 min to promote wetting of the pores. After vacuum treatment, the functionalized membranes were rinsed with fresh toluene and cured for 2 h at 80 °C.^32^

The amine densities of NH_2_–AAO (amine functionalized AAO membranes) were quantified using a colorimetric assay with acid orange II (AO II), an anionic dye. NH_2_–AAO was submersed in a 5 mL aqueous solution of 0.5 mM AO II, adjusted to pH 8 with NH_4_OH, placed under vacuum for 30 min, and incubated on a shaker plate with moderate agitation for 90 min. Unbound AO II was then rinsed from the membranes with fresh pH 8 solution. Bound AO II was released into 5 mL of pH 12 solution. The concentration of AO II in the resulting solution was measured by UV-vis at 495 nm with an Agilent BioTek Synergy H1 Plate Reader (see Fig. S1 for the calibration curve).^33^ Full release of bound AO II at pH 12 was confirmed by the UV-signal returning to the background level after the second wash. The amount of AO II released was measured from triplicate experiments (three independent membranes) from the combined AO II released from the two desorption solutions.

Heparin was coupled to the primary amines on the surface of NH_2_–AAO using carbodiimide chemistry. NH_2_–AAO was incubated in 20 mL of 0.1 M MES buffer with 1.6 mg per mL EDC, 0.96 mg per mL NHS, and 76 µg per mL heparin overnight with moderate agitation.^34^ The membranes were initially placed under vacuum for 30 min to fully wet all surfaces. Following heparinization, the membranes were rinsed with UF H_2_O, dried, and stored at 4 °C.

Heparin density on HEP–AAO (heparinized membranes) was quantified using methylene blue (MB), a cationic dye, which was adsorbed and desorbed by heparin through electrostatic interactions with sulfate groups.^35^ Binding took place in 5 mL of a 0.16 mM MB solution (pH 6) over 2 hours. Bound dye was eluted for 30 min through charge screening into 5 mL of a 0.5 M NaCl solution.^36^ The concentration of MB in the eluted dye solution was measured using UV-vis at 668 nm using a standard curve (Fig. S3).

Primary amines on NH_2_–AAO and HEP–AAO were capped with a terminal carboxyl group by forming an amide bond with citraconic anhydride using a ring opening mechanism.^37^ Citraconic anhydride (1% v/v) was added dropwise to 0.1 M sodium carbonate–bicarbonate buffer (pH 9). The AAO was placed in 5 mL of solution and incubated for 30 min, before being placed under vacuum for 30 min. Following the reaction, the RCOO–NH_2_–AAO/RCOO–HEP–AAO (where RCOO indicates amine capping) membranes were rinsed with fresh buffer, H_2_O, and then ethanol before drying at 80 °C. The density of uncapped amines was quantified using AO II, as described above.

##### Synthesis of FITC-dyed 20 nm silica nanoparticles (SNPs)

SNPs (nominally 20 nm diameter) were synthesized using the Stöber method, with reagent amounts calculated through correlations developed by Bogush *et al.*^38^ In a round bottom flask, 9 mL TEOS and 2.2 mL NH_4_OH were added to 88.8 mL anhydrous ethanol, with TEOS added in a rapid dropwise manner using a separatory funnel. The solution was allowed to react overnight. To induce aggregation,^39^ 100 µL of an aqueous MgCl_2_ (50% w/w) solution was added to 100 mL SNP solution and the SNP-salt solution was aged for 2–3 h. Particles were then recovered *via* centrifugation. The recovered particles were rinsed thrice with ethanol, prior to curing at 200 °C overnight. The SNPs were then desalted using regenerated cellulose (RC) tubing with a MWCO of 3.5 kDa (Spectra/Por) and dried at 100 °C overnight.

SNPs were hydroxylated to saturate their surfaces with hydroxyl groups prior to amine functionalization.^40^ Particles (0.5 mg mL^−1^) were submersed in a 50/50 v/v methanol/H_2_O solution, adjusted to pH 3 with nitric acid. The particles were dispersed using sonication and incubated in the solution at 40 °C for 4 h with moderate agitation. The SNPs were then recovered *via* centrifugation, rinsed twice with anhydrous ethanol, resuspending in between washes, and dried overnight at 80 °C.

Hydroxylated SNPs were amine functionalized in anhydrous ethanol at 0.5 mg mL^−1^. Under nitrogen, APTES was slowly added drop-wise to a final concentration of 0.5% v/v.^40^ The reaction was allowed to proceed overnight with moderate agitation. SNPAs (amine functionalized SNPs) were recovered through centrifugation, washed twice with anhydrous ethanol, and cured at 80 °C overnight.

The amine density on SNPAs was quantified through a colorimetric assay using AO II.^33^ SNPAs (2.5 mg mL^−1^) were added to an aqueous 0.5 mM AO II solution, adjusted to pH 3 with HCl. SNPAs were incubated in the dye solution for 2 h, recovered by centrifugation and rinsed with aqueous pH 3 solution. The dye was released using pH 12 solution. The dye release step was repeated two to three times until all measurable AO II was desorbed. The concentration of AO II in each supernatant was quantified at 495 nm using a standard curve.

FITC was covalently bound to the amines on the SNPA surface.^40^ SNPAs were dispersed (0.5 mg mL^−1^) in 0.0642 mM FITC in anhydrous ethanol. The reaction was allowed to proceed overnight. The FITC–SNPs (FITC dyed SNPAs) were recovered by centrifugation, rinsed with ethanol twice, and dried overnight at 80 °C.

##### Characterization of SNPs, SNPAs, and FITC–SNPs

SNPs were prepared for scanning electron microscopy (SEM) by suspending a small amount in anhydrous ethanol or UF H_2_O, depositing onto a silicon wafer which was plasma-cleaned with a Leica ACE 600, and drying overnight under vacuum. The dried sample was then coated with a thin layer of platinum (0.5–2 nm) using a Leica ACE 600 and imaged using a FEI Helios Nanolab 660. ImageJ was used to measure particle diameters.

The hydrated diameters of SNPs were measured through dynamic light scattering (DLS) with an Anton Paar Litesizer 500. Particles (0.5–0.8 mg mL^−1^) were suspended in UF H_2_O with sonication and vortexing to ensure particle dispersion.

Zeta potential (*ζ*) measurements were conducted in triplicates on SNPs, SNPAs, or FITC–SNPs (0.5 mg mL^−1^) to confirm surface charges consistent with functionalization. SNPs, SNPAs, or FITC–SNPs were suspended in aqueous solutions at pH 3.1, pH 5.0, pH 8.3 and pH 11.9. The pH of the aqueous solutions was adjusted with HCl or NH_4_OH. The solution was then pipetted into an omega cuvette and analyzed with an Anton Paar Litesizer 500.

##### Bioaffinity assay by protein binding

The static capture and release of FGF2, quantified using the fluorescence of reporter antibody-associated protein,^41–43^ was used to infer the presence of bioactive heparin on HEP–AAO. To bind the protein, HEP–AAO, NH_2_–AAO (control) and AAO (control) were incubated in 3 mL of a 0.33 µM FGF2 in 5 mM Tris–HCl solution for 4 h at room temperature. The membranes were rinsed with fresh buffer prior to being incubated in 3 mL of an equimolar solution of the FITC conjugated antibody of FGF2 (Ab-FITC) overnight. The membranes were then rinsed with fresh buffer and placed in 3 mL of 2 M NaCl in 50 mM Tris–HCl for 2 h to elute the protein and antibody from the membranes. The concentration of the fluorescent reporter antibody-associated protein released in the resulting solutions was quantified using a standard curve constructed from excited at 490 nm and emission measured at 520 nm (Fig. S5). The binding and release of FGF2 was interpreted assuming a 1 : 1 binding with Ab-FITC. The effect of amines on nonspecific binding of FGF2 was investigated using amine-blocked RCOO–NH_2_–AAO and RCOO–HEP–AAO membranes using the methods described above. The pH was adjusted to 8 to prevent the reversal of amine blocking^37^ and the extent of FGF2 was uptake and release was quantified using a standard curve constructed at pH 8 (Fig. S6).

##### Static capture of 20 nm FITC–SNP

Pore accessibility and membrane capacity of bare and functionalized AAO was assessed using electrostatic capture of 20 nm FITC–SNP on HEP–AAO (with NH_2_–AAO as a control). Prior to particle uptake, membranes were preconditioned by soaking in 10 mL of aqueous solution at pH 3 for 10–15 min under vacuum. Membranes were then submersed in 10 mL of a 0.2 mg mL^−1^ FITC–SNP aqueous dispersion at pH 3. Following a 30 min vacuum treatment, the membranes were incubated in the particle solution overnight. Unbound FITC–SNPs were rinsed from the membrane using 10 mL of pH 3 solution, assisted by brief agitation and vacuum treatments. FITC–SNPs were desorbed from the membranes by charge screening using 10 mL of aqueous 1 M NaCl solution. Sonication and vacuum treatments were used prior to overnight incubation in the NaCl solution to maximize particle desorption. The concentration of FITC–SNP in the eluants was analyzed with excitation/emission at 490/520 nm and quantified using a standard curve (Fig. S8). Capture experiments were completed in triplicate using three independent membranes.

##### Dynamic capture of AAV2

The capture and release of AAV2 on HEP–AAO was investigated in tangential flow filtration (TFF) mode. The TFF apparatus consisted of a 47 mm stainless steel Pioneer TFF cell (Model CF047, Sterlitech Corp.) with a 25 mm membrane adapter (which held the HEP–AAO), and peristaltic pump. All equipment was operated in a biosafety cabinet and under biosafety level BSL2 safety protocols. Prior to this experiment, AAV2 cell lysate in PBS (pH 6) underwent depth filtration and ultrafiltration to remove the majority of cell debris and other impurities (see Fig. 1), as described by Leach *et al.*, resulting in a final AAV2 titer of 1.51 ± 1.8 10^11^ capsids per mL.^30^ To capture AAV2, approximately 50 mL of the feed (in pH 6 PBS) was filtered through a HEP–AAO membrane (capture permeate) to achieve a minimum of 35 L m^−2^ per manufacturer specifications. The flow rate was approximately 30 mL min^−1^ with a pressure drop of 0.1–0.5 bar for this and subsequent rinse and release steps. Flux data were recorded using a Mettler Toledo BalanceLink.^44^ Following the AAV2 capture step, the membrane was rinsed with 50 mL of fresh pH 6 PBS to remove any unbound viral vectors and other impurities. Once the HEP–AAO was rinsed, bound AAV2 was released by performing TFF with approximately 50 mL of 1 M NaCl solution (eluent). The concentration of AAV2, DNA, and protein in the feed, permeate during capture, and rinse eluent were quantified using ELISA, a bisBenzimide DNA Quantification Kit, and a Bradford Protein assay, respectively, using methods described by Leach *et al.*^30^ Standard curves for the quantification of AAV2, DNA and protein are provided in Fig. S10–S12.

## Results and discussion

### AAO surface functionalization

AAO membranes were selected for this investigation, hypothesizing that the densely packed pores with smooth surface and low tortuosity would provide a unique environment to explore the capture and release of biocolloids relative to traditional polymer membranes.^45^ In addition, the rigid non-swelling structure of AAOs allows for the retention of selectivity.^46^ The top surface and cross sectional areas of the commercial AAO supports (21 mm active diameter and 60 µm thick) are shown in SEM images of a representative AAO supports (Fig. 2). The pore diameter, analyzed from Fig. 2a using ImageJ software and automation, was 224 + 33 nm (*n* = 200), in agreement with the reported value from the manufacturer. Cross sectional images (Fig. 2b) confirm straight, uniformly sized pores that span the entire thickness of the membrane.

**Fig. 2:**
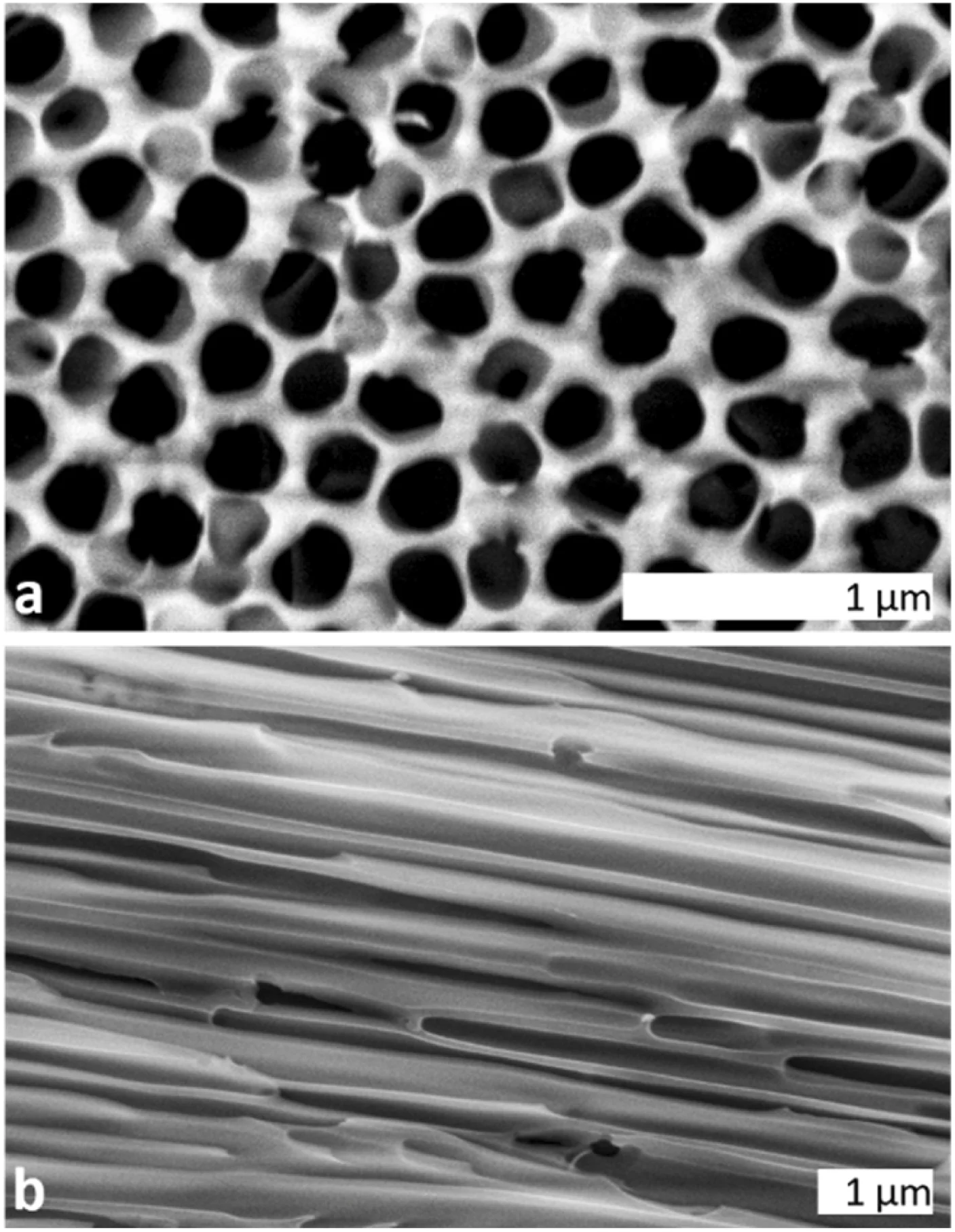
SEM images of base AAO membrane. (a) Surface view of a membrane showing uniform, open channels and (b) membrane cross section showing straight, non-intersecting pores.

Membrane porosity was determined from measured flux of UF water over a pressure drop range from 30–60 kPa. The uniform cylindrical pores of AAO are consistent with the assumptions of the Hagen–Poiseuille equation, which was rearranged (eqn (1)) to determine the membrane porosity from the measured flux (*J*) and transmembrane pressure drop (Δ*P*):1
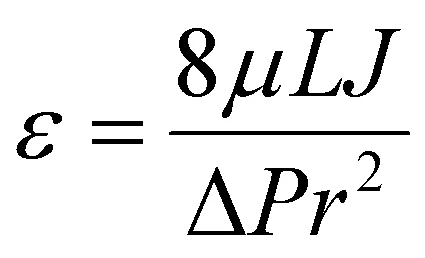
where *ε* is the membrane's porosity, *µ* is the solvent viscosity, *L* is the membrane thickness, and *r* is the pore radius. From linear plots of *J versus* Δ*P* (Fig. 3, *n* = 3 membranes) the porosity was determined to be 0.636 + 0.17. This is higher than the porosity range reported by the manufacture (0.25–0.5) but consistent with the area fraction of pores visualised in Fig. 2a.

**Fig. 3:**
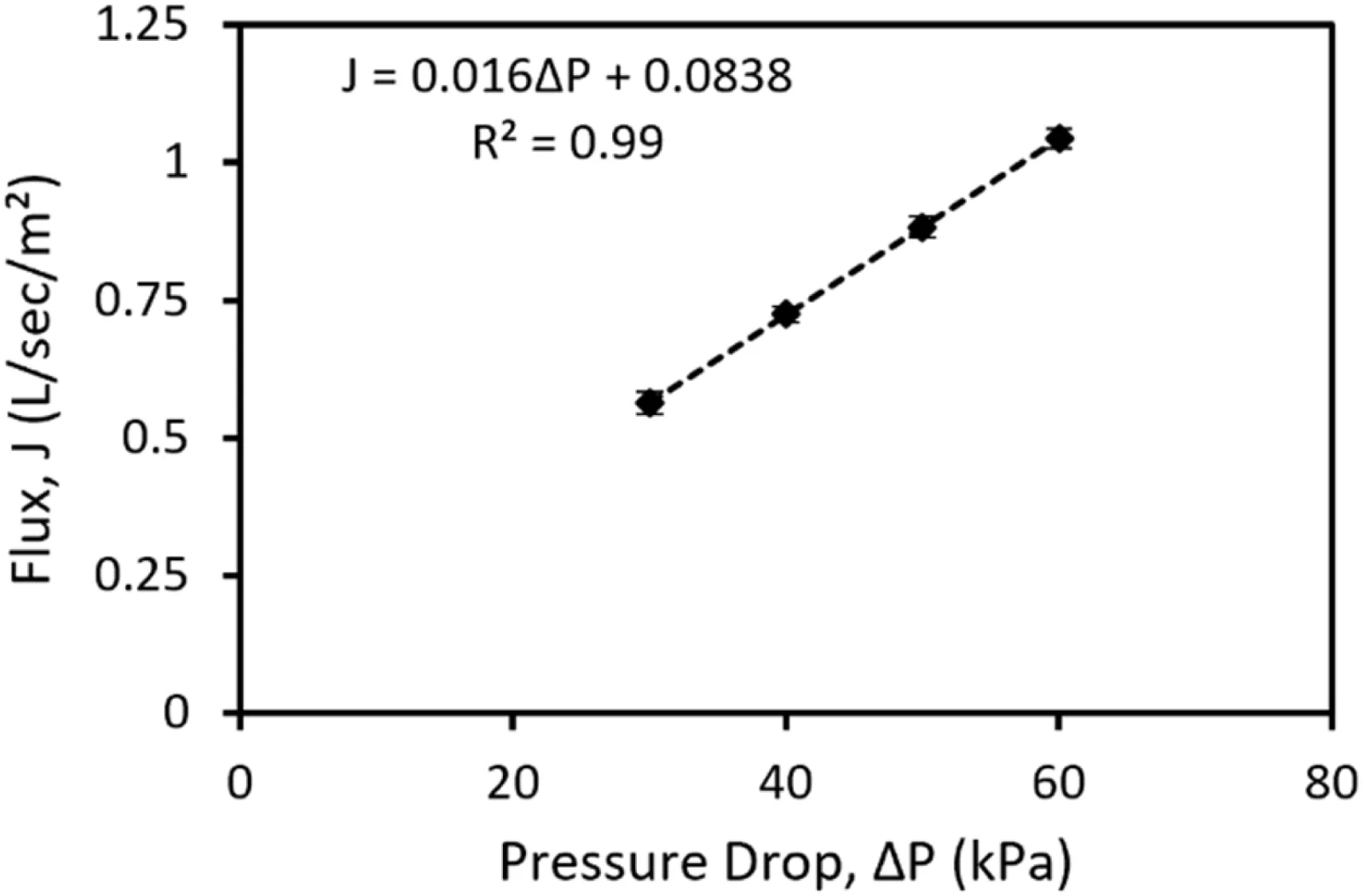
Flux profile of deionized water through AAO membranes as a function of pressure drop across the membrane. The error bars represent standard deviations (*n* = 3 independent membranes with 6–8 flux measurements per membrane).

The surface area of the membrane was calculated using the manufacturer reported values of membrane active diameter and thickness, the measured pore diameter, and the experimentally obtained porosity. This resulted in a total surface area of 0.240 + 0.06 m^2^, assuming all pores are uniform, cylindrical, and open on both ends. The surface area is dominated by the interior pore surface (99%).

The extent of functionalization of the AAO membranes with accessible amines was characterized using the anionic dye AO II and assuming its 1 : 1 binding with protonated primary amines.^33^ AO II was loaded onto protonated amines at pH 8, alumina's isoelectric point (IEP), to prevent the interaction of the dye with the surface of the membrane.^47^ AO II was released and collected in a pH 12 solution, which deprotonated the amines. An intense orange color is observed in the membranes after AO II loading at pH 8 (Fig. S2a) and the color is removed at pH 12 (Fig. S2b). Across amine-functionalized AAO supports, the density of accessible amines was 0.81 ± 0.6 µmol m^−2^. This corresponds to an average of 25% of a monolayer, where a monolayer of amines is expected to have a density of 3.3 µmol m^−2^, assuming an area per aminopropylsilane of 0.5 nm^2^.^48^

Following amine attachment, heparin (HEP) was conjugated to the surface by EDC–NHS coupling *via* the carboxylic acid groups of HEP (Fig. 4). To quantify the attachment of accessible heparin, the cationic dye methylene blue (MB) was bound and released from the sulfate groups of HEP.^49^ MB was loaded at pH 6 (which was previously reported not to interfere with MB/HEP electrostatic interactions^49^) and released by into aqueous 0.5 M NaCl by charge screening. The membranes become intensely blue with MB bound (Fig. S4a), and that color is lost upon exposure to salt solution (Fig. S4b). The amount of MB released was converted to an equivalent mass of heparin by assuming that the HEP sulfate groups were saturated with MB, bound 1 : 1, and that the structure of HEP could be described as uronic acid–glucosamine building blocks comprising three sulfate groups (MW = 595). The accessible heparin density on membranes was 1.05 ± 0.21 mg m^−2^. For reference, the uptake of MB by NH_2_–AAO is below the detection limit.

**Fig. 4:**
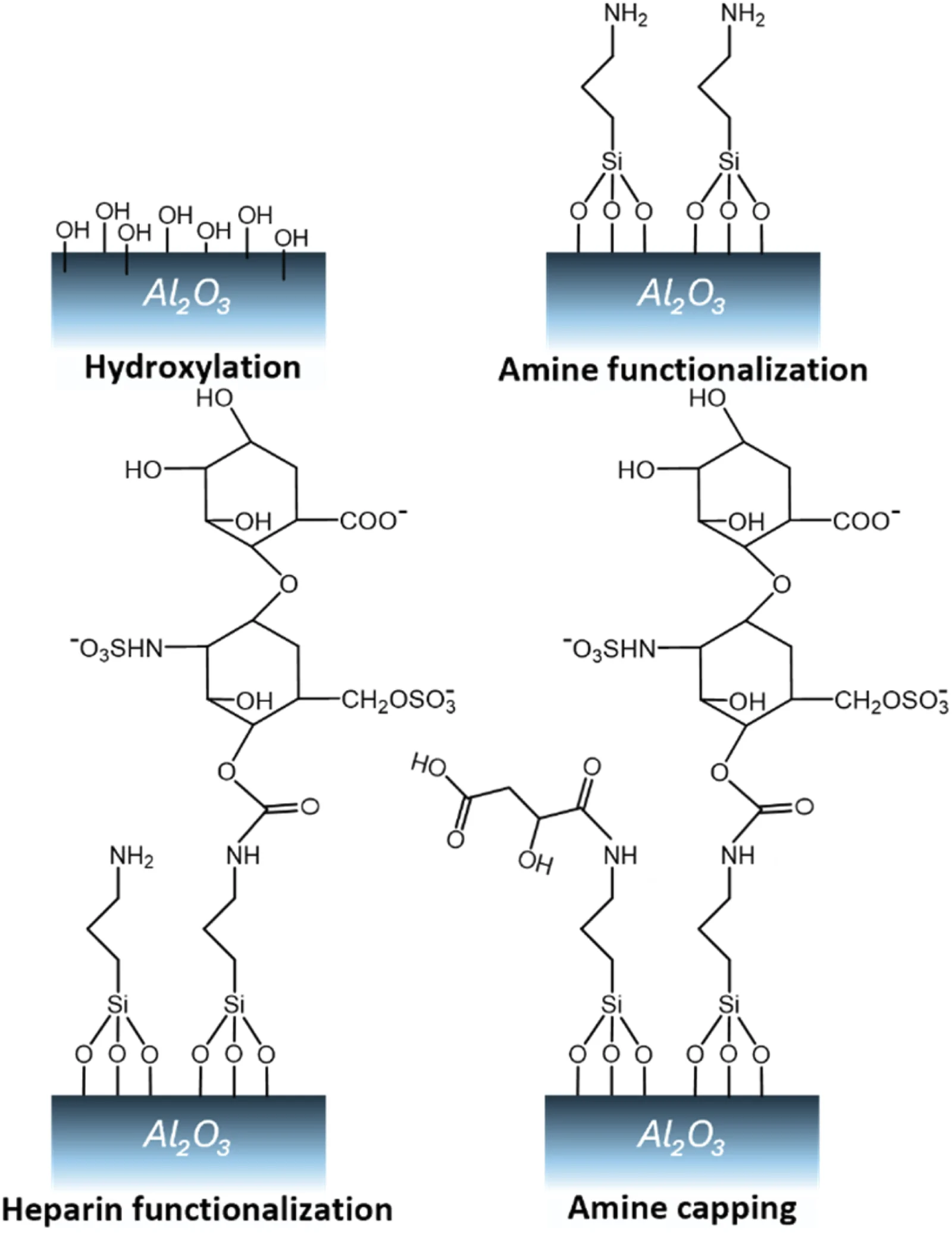
Schematic of the steps of functionalization of the AAO surface. Hydroxylation first saturates the surface with hydroxyl groups. APTES was used to attach primary amines to the surface. Heparin was then attached to the amine-functionalized surface through formation of a carbodiimide bond. Residual free amines were capped on amine-functionalized and heparin functionalized supports using citraconic anhydride to form a terminal carboxylate group.

In the investigation of the affinity binding of FGF2 to immobilized heparin, residual amines on NH_2_–AAO and HEP–AAO were blocked using citraconic anhydride with a goal of reducing nonspecific binding. Citraconic anhydride reacts with primary amines to form an amide bond by a ring opening mechanism, leaving a terminal carboxylic acid (Fig. 4). This reaction is reversed when blocked amines are exposed to acidic conditions, causing the hydroxylation of the amide bond and subsequently releasing the citraconic anhydride. However, this bond is stable above pH 8.^37,50^ Amine densities on NH_2_–AAO and HEP–AAO were measured before and after blocking with citraconic anhydride using AO II. As an example of the effect of amine capping, the amine density of an NH_2_–AAO membrane with 0.422 µmol NH_2_ per m^2^ was reduced 84% in the corresponding RCOO–NH_2_–AAO.

### Affinity uptake of proteins

The bioaffinity of immobilized heparin was analyzed through the capture and release of fibroblast growth factor (FGF2), as quantified using a fluorescent reporter antibody. FGF2 is a heparin-binding protein that is involved in a variety of biological pathways, such as inflammation responses, as well as organ and cell growth and development.^51^ Heparin binds to FGF2 through specific interactions of the sulfates in heparin and the amino acid sequences in FGF.^26^ The FGF2 binding demonstrates the affinity of immobilized heparin, and was compared to bare and amine-functionalized AAO as controls. In these experiments, membranes were exposed to FGF2 in Tris buffer, followed by binding of FITC-conjugated antibody to FGF2. After washing, bound protein was released into 2 M NaCl Tris buffer and quantified using a calibration curve (Fig. S5). The fluorescence emission spectra (Fig. 5a) show significantly more FGF2 was bound to HEP–AAO than the controls. HEP–AAO released 0.551 nmol of FGF2, 61.2% of initially available growth factor. Conversely, NH_2_–AAO and AAO only bound 0.342 nmol and 0.0329 nmol of FGF2, 38.0% and 3.7% of FGF2 initially available, respectively.

**Fig. 5:**
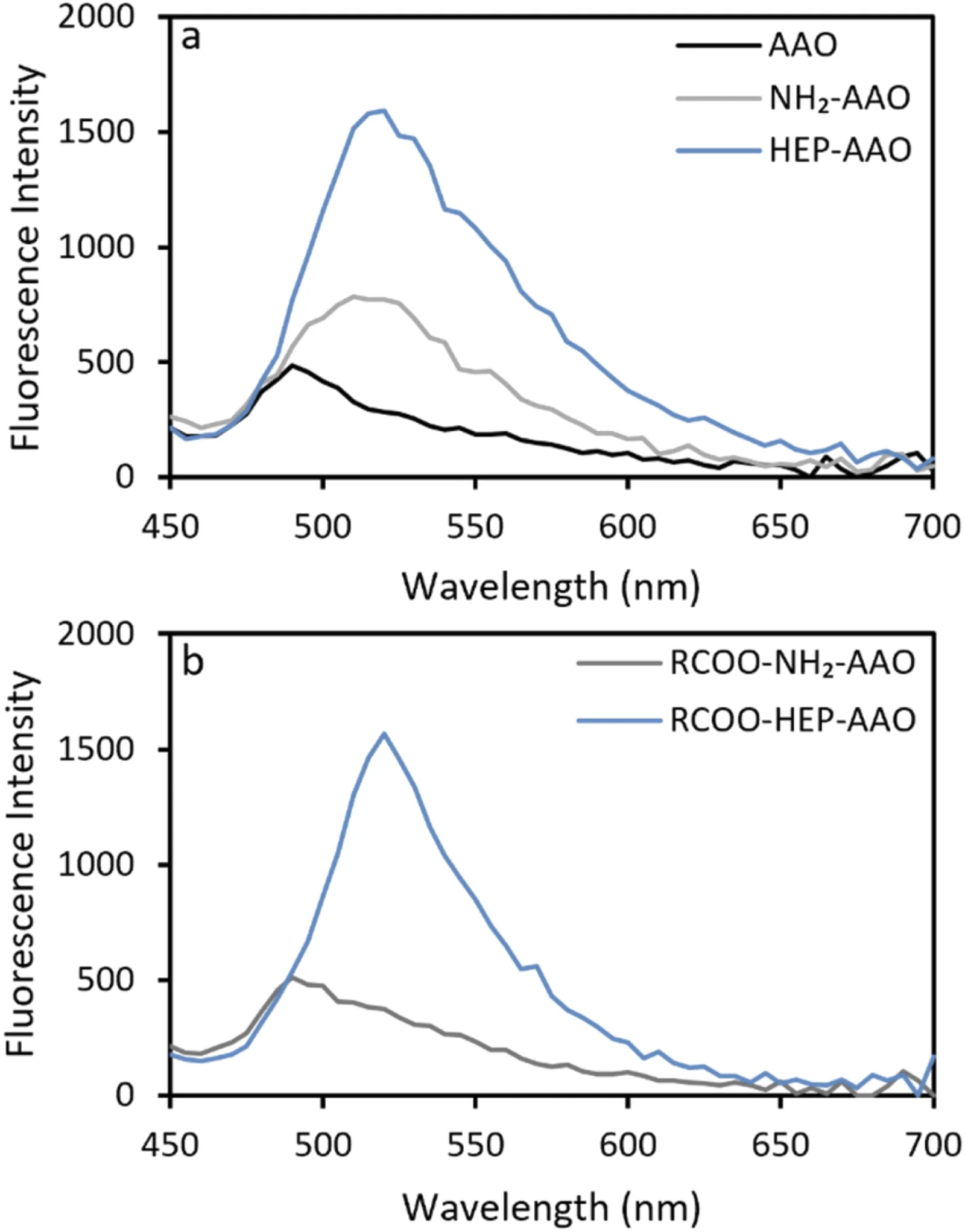
Fluorescence emission spectra of FGF2 Ab-FITC released from bioaffinity experiments. (a) Is the spectra of protein and antibody captured by AAO, NH_2_–AAO, and HEP–AAO and released in 2 M NaCl in 50 mM Tris–HCl. (b) Is the spectra of the protein and antibody captured on functionalized amine-capped membranes, RCOO–NH_2_–AAO and RCOO–HEP–AAO, and released in 2 M NaCl in 50 mM Tris–HCl, with the pH adjusted to pH 8 to prevent the reversal of the amine capping.

The significant FGF2 uptake by the NH_2_–AAO control was hypothesized to be due to nonspecific binding with the positively charged membrane surface. To uncouple the bioaffinity of FGF2 for heparin from its nonspecific interactions with the amine functionalized surface, the primary amines of NH_2_–AAO and HEP–AAO membranes were blocked with citraconic anhydride and the uptake experiments were repeated. The pH was shifted to pH 8 to prevent the reversal of amine capping. Due to the pH shift, a new standard curve was made (Fig. S6). The amount of FGF2 captured by RCOO–HEP–AAO (0.157 nmol) was reduced relative to the uncapped HEP–AAO. However, the binding of FGF2 to the corresponding control (RCOO–NH_2_–AAO; (0.0230 nmol)) was also reduced relative to uncapped NH_2_–AAO, and the binding roughly corresponded to the background binding of the bare AAO support. Overall, the nonspecific binding of FGF2 by RCOO–NH_2_–AAO was reduced to 15% of the amount captured by RCOO–NH_2_–AAO (Fig. 5b).

### Electrostatic uptake of virus-sized particles

Silica virus-sized particles were used to investigate the accessibility, capacity and reusability of 200 nm pore diameter AAO membranes for the capture of 20 nm diameter biocolloids. SNPs are used widely in the biomedical and pharmaceutical industries for applications including imaging and drug delivery.^52,53^ Monodisperse, nonporous SNPs were synthesized by the Stöber method^54^ using the correlation of Bogush *et al.*^38^ to achieve the desired particle size. The resulting particles are roughly spherical with a diameter of 16.1 ± 5.1 nm (*n* = 1200) (SEM image provided in Fig. S7a). From dynamic light scattering, the hydrated diameter of the SNPs was 29.3 nm (Fig. S7b), giving an estimated range consistent with the reported size of AAV biocolloids.^1^

SNPs were then functionalized with amines (SNPA) to provide for electrostatic interactions with immobilized HEP on AAO supports. AO II was used to quantify the amine density of SNPA, using a procedure similar to that used for AAO membranes. The accessible amine density was determined to be 1.74 + 0.078 µmol m^−2^ (*n* = 8 independent samples of SNPA) or roughly 53% of monolayer coverage of the amines on the silica surface. Following amine functionalization, FITC was conjugated to the surface to facilitate quantification SNPs in capture and release experiments with AAO membranes.

The zeta potentials (*ζ*) of 20 nm SNPs, SNPAs, and FITC–SNPs were measured as a function of pH (Fig. 6). The bare SNPs were found to have a negative surface charge consistent with the presence of deprotonated silanols. They exhibit a nearly neutral charge around pH 3, the IEP of silica, consistent with previous reports.^55^ The functionalization of SNPs with amines results in the expected shift to positive zeta potential, with an IEP of approximately pH 6. Covalent attachment of FITC to the SNPAs had a minimal impact on the corresponding zeta potential, meaning that surface density of the amines was not significantly changed. The FITC–SNPs are roughly neutral at pH 6, mimicking the charge of AAV2, as empty and full capsids have IEPs of 6.3 and 5.9, respectively.^56^ The *ζ* results suggest that SNP–FITC should interact most strongly with sulfates of HEP at low pH and should be released at pH > 6, as the particles become neutral and then negatively charged with increased pH.

**Fig. 6:**
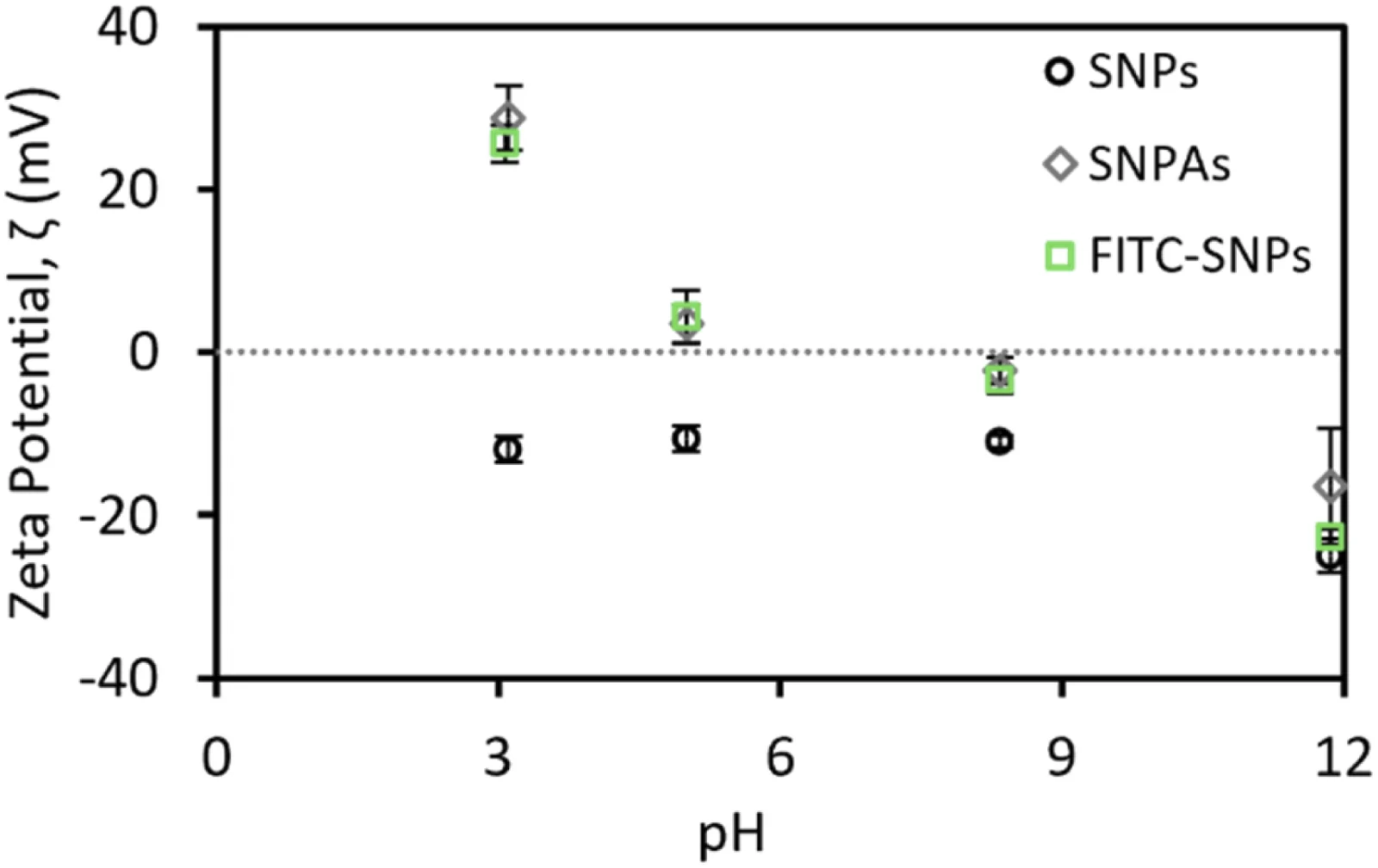
Zeta potential measurements of 20 nm SNPs. Measurements of 20 nm SNPs, SNPAs, and FITC–SNPs were conducted in triplicate over a range of pHs to analyze the change in surface charge.

Static membrane uptake and release experiments were used to demonstrate electrostatic binding and release of 20 nm particles in functionalized AAO and the accessibility of the approximately 200 nm AAO pore structure. FITC–SNPs were adsorbed at optimal conditions for HEP–AAO (adsorption at pH 3) and released by charge screening into a 1 M NaCl solution (a calibration curve is provided in Fig. S8). NH_2_–AAO served as a control to differentiate uptake by electrostatic interactions from nonspecific and size-based interactions. The recovery of FITC–SNPs from three cycles of binding and release were compared to demonstrate the reversibility of the electrostatic interactions and the potential reuse of the membrane support (Fig. 7). The membranes were soaked in pH 3 solution to remove residual salt (after elution of bound particles) between cycles of binding and release.

**Fig. 7:**
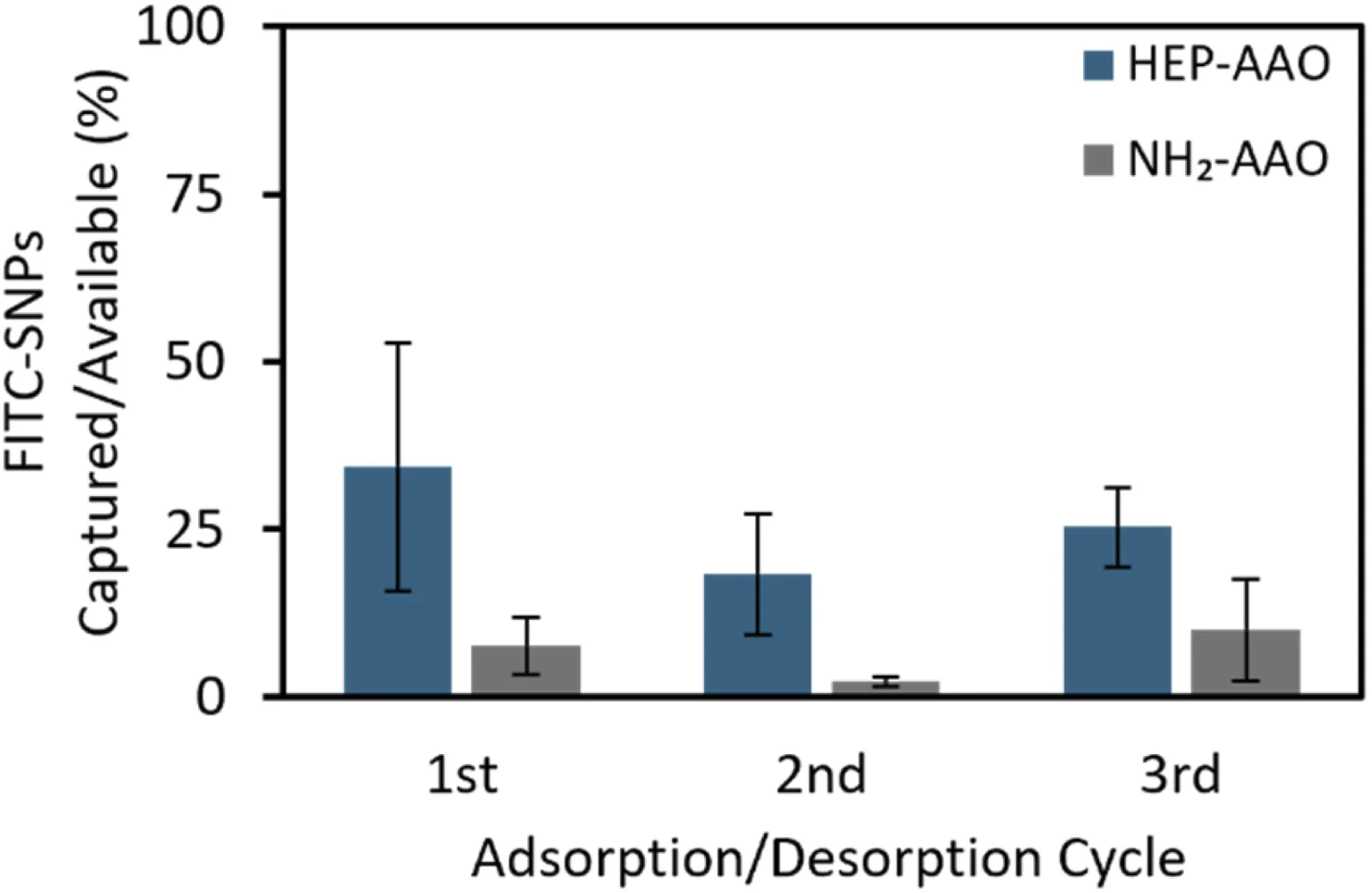
Recovery of 20 nm FITC–SNP from three cycles static adsorption/desorption on HEP–AAO and NH_2_–AAO membranes to demonstrate reusability of the membranes. Percent bound based on FITC-SNPs initially available to the membrane. The error bars represent standard deviations (*n* = 3 independent membranes).

Uptake of the FITC–SNPs at pH 3 gave a visible yellow color to the membranes which was lost after release. Assuming that the maximum amount of FITC–SNPs that can be captured would be in a hexagonal close packed (HCP) adlayer, the theoretical capacity of a 21 mm AAO membrane with 224 nm pores and 64% porosity was determined to be 938 µg (see Fig. S9 and corresponding discussion). Heparinized membranes captured a mean value of 700 µg for the 1st cycle (equivalent to 1.6 × 10^14^ particles) or 73% of theoretical capacity. To give a sense of how membrane modules would need to scale for affinity capture, this translates to the capture of 4.7 × 10^17^ particles per superficial membrane m^2^.

The mean capacity of HEP–AAO appeared to decrease after the first use (Fig. 7), but there was not a statistically significant difference between the 1st, 2nd, or 3rd cycle of the FITC–SNP binding experiments based on ANOVA analysis. This was true for both the HEP–AAO and NH_2_–AAO series. However, a *t*-test applied to the combined data from all three cycles revealed that there is a statistically significant difference (*p* = 0.00071) between HEP–AAO (26 ± 13% captured/available) and NH_2_–AAO binding (6.6 ± 5.6%). The background binding by NH_2_–AAO fluctuates and can be significant (mean value = 6.6% of available FITC–SNP). We hypothesize that this background binding could be a reduced by capping the amines on the AAO supports, as was done for the FGF2 binding experiments.

### Dynamic capture of AAV2

To demonstrate the capture of viral vectors on HEP–AAO membranes under dynamic conditions, AAV2 was captured and released from clarified cell lysate under tangential flow filtration (TFF). Chaubal *et al.* recently demonstrated the use of single pass tangential flow filtration (SPTFF) for the concentration of AAV from clarified cell lysate.^57^ In SPTFF, AAV was concentrated in the membrane retentate by a filtration mechanism and the permeate containing the impurities passed through the membrane. This is consistent with previous investigations of the concentration of biologics using TFF using submicron sized pores in filtration mode.^58^ Alternatively, TFF using membranes with micron-sized pores can be used to clarify the cell lysate by allowing the biologic to pass through the cell membrane as the permeate and the cell debris removed in the retentate.^59^

When applying TFF using the 224 nm pore diameter HEP–AAO, flow through the membranes was used to promote capture and release of the 20 nm viral vectors on the pore walls. The system was operated at a maximum transmembrane pressure of 0.5 bar. The affinity capture of AAV2 is envisioned as a final step in a series of membrane processes with an input of AAV2 cell culture that has been pre-treated to maximize the affinity capture (Fig. 1). The feed to the process was AAV2 cell culture lysate that was clarified by depth filtration and ultrafiltration to remove cell debris and some of the residual protein and DNA in the solution.^30^

AAV2 was captured by membrane-bound heparin ligand from the clarified AAV2 lysate as the lysate flowed through the on HEP–AAO membrane in TFF mode. The pH of clarified AAV2 solution was adjusted to pH 6 to maximize the interactions of AAV2 with HEP–AAO, as suggested by *ζ* potential measurements. During TFF, the solution passing across the membrane (referred to as “capture permeate”) was collected for further analysis. Following the capture step, pH 6 buffer was passed through the membrane to remove unbound AAV2 and impurities. Finally, 1 M NaCl was passed through the membrane to release bound AAV2. The eluent from the release step was collected for further analysis. Fig. 8 illustrates the steps used to capture, rinse, and release AAV2 from the HEP–AAO membrane.

**Fig. 8:**
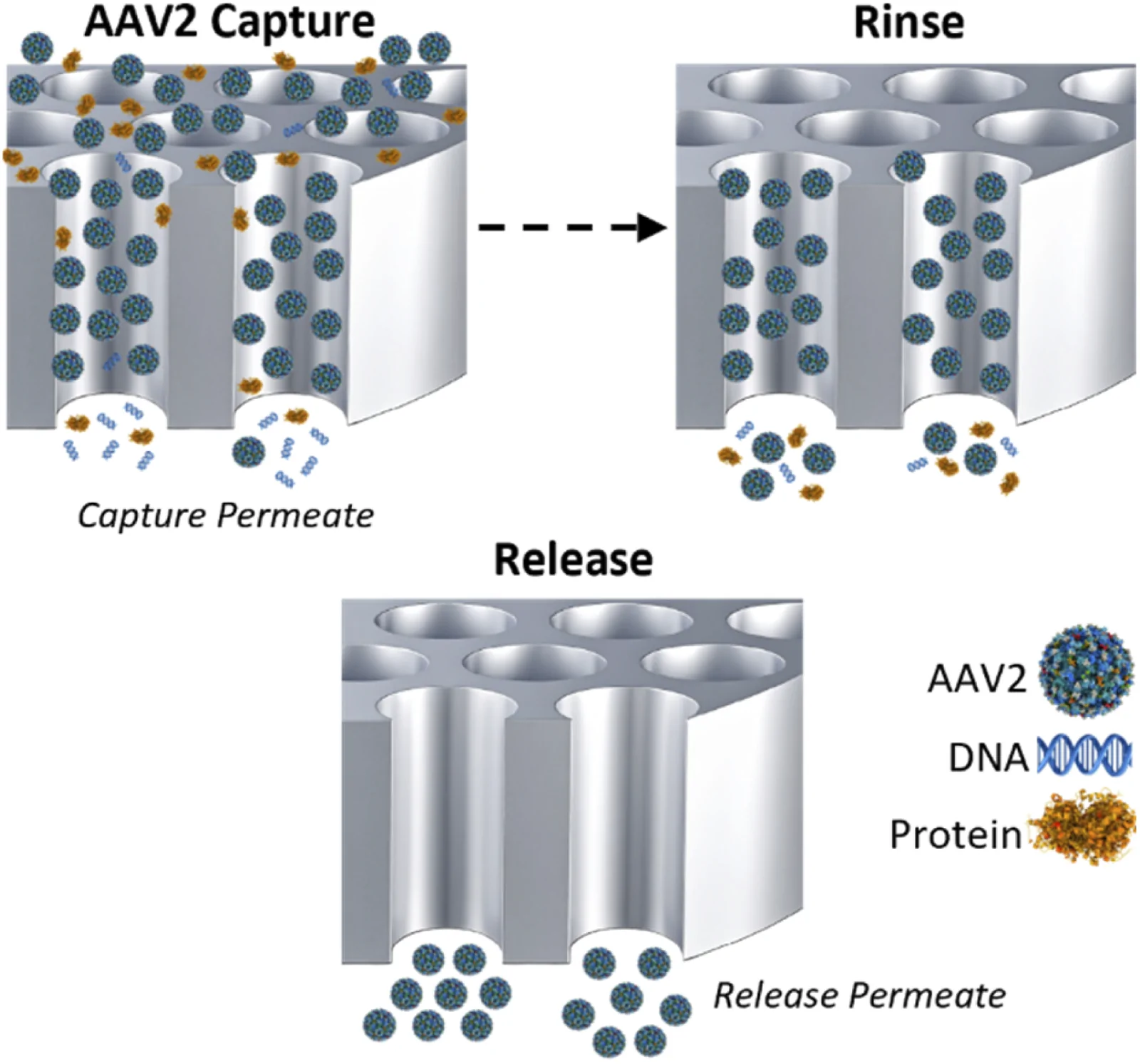
Capture, rinse, and release of AAV2 from HEP–AAO under TFF. AAV2 cell lysate was captured in PBS (pH 6), resulting in the capture permeate. Unbound AAV2 and biological impurities (protein and DNA) are removed by rinsing with PBS (pH 6). Releasing bound AAV2 from HEP–AAO using 1 M NaCl forms the release permeate.

The flux profiles across the membrane in the TFF cell during the capture, rinse and release steps are presented in Fig. 9. The startup profile is variable as the flow increases while the system reaches the desired pressures of operation. However, the later stages of operation can be used to interpret fouling. A high throughput was observed in each step, suggesting negligible fouling.

**Fig. 9:**
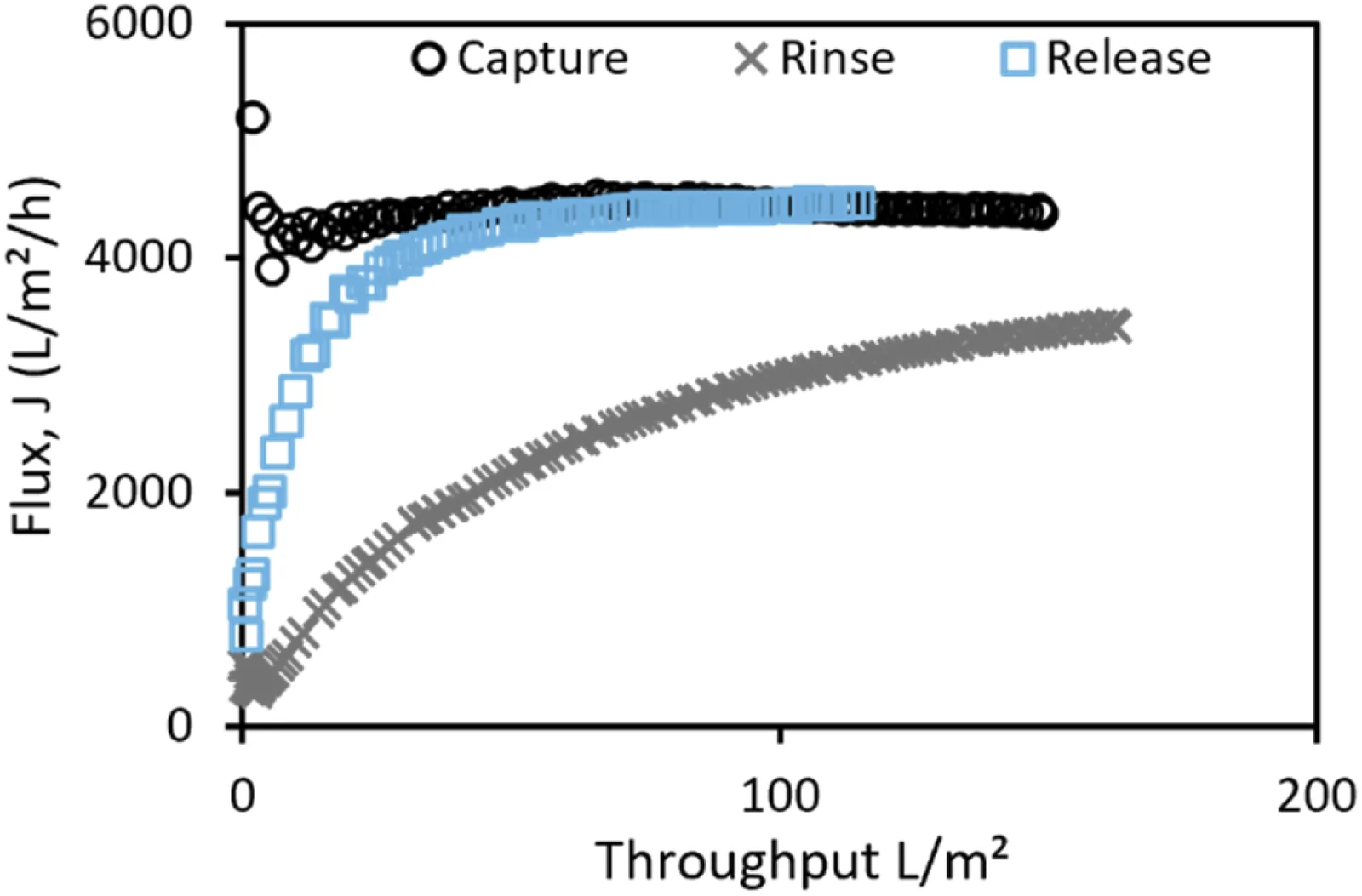
TFF flux profiles of HEP–AAO membranes. Flux measurements were taken during the capture, rinse, and release of AAV2 by HEP–AAO.

The permeate concentrations of AAV2, DNA, and protein during the capture step and eluent concentrations from the recovery step are compared to the initial feed in Table 1. During the capture step, 99% of AAV2 was removed from the pre-processed AAV2 solution by HEP–AAO, with a 25% and 93% reduction in host DNA and protein, respectively. The release step with 1 M NaCl contained an AAV2 titer that was 70% of the initial feed titer, indicating that most AAV2 captured in the capture step remained bound to the heparin during the rinse step of the membrane. Furthermore, protein and DNA were not detected in the eluent of the release step, suggesting that the rinse step was successful at removing unbound biological molecules. This level of AAV recovery compares favorably to levels reported in the literature (65–80%) for commercial heparin affinity chromatography under optimized conditions.^60^ Based on the pore diameter and porosity, the amount of AAV2 captured is 2.12 × 10^13^ capsids per m^2^, or 2.1% of the measured SNP capacity, providing the opportunity to increase AAV2 capture.

**Table 1:** Concentrations of AAV2, BSA, and DNA in the feed, capture permeate, and release permeate measured during affinity membrane capture using HEP–AAO. Feed concentrations were determined from triplicate measurements. Dynamic capture was performed using 43.4 mL of feed (corresponding to the volume of feed and capture permeate). The release step used 37.7 mL of feed (release permeate volume) approximate detection limits for protein and DNA are 0.21 mg mL^−1^ and 0.0053 µg mL^−1^, respectively

Sample	AAV2 capsids (10^12^)	Protein (mg)	DNA (µg)
Feed	6.5 ± 1.8	14 ± 1	1.74 ± 0.08
Capture permeate	0.021	0.96	1.30
Release permeate	4.6	Below detection	Below detection

Unlike polymeric membranes, AAOs have low tortuosity and a highly ordered structure, which contribute to their anti-fouling properties. In ultrafiltration experiments performed by Sharma *et al.*, an alumina membrane was evaluated against a comparable polymeric membrane in filtering a model viral vector solution of protein nanoparticles based on size-exclusion.^61^ The alumina membrane not only had higher fouling resistance to biologics compared to the polymeric membrane, but the majority of its permeability was restored through rinsing with buffer, while the polymeric membrane required further chemical cleaning.^61^ In the previously mentioned work of Wang *et al.*, a heparinized cellulose membrane was used to capture recombinant adeno-associated virus serotype 2 (rAAV2) through static capture.^29^ At pH 6, this membrane captured about 20% of available rAAV2.^29^ Bartels *et al.* further demonstrated the ability of ceramic membranes to be easily regenerated during virus separations, successfully regenerating yttria-stabilized zirconia capillary membranes during separation of MS2 bacteriophages through backflushing, which had no impact on the continued performance of the membrane.^46^

## Conclusions

In this work, we developed synthesis and characterization strategies for affinity membranes suitable for separation and purification of therapeutic biocolloids using heparinized AAO supports. Surface functionalization strategies were used to tune the electrostatic binding and release of virus-sized SNPs (20 nm diameter model systems of AAV2) from these ceramic supports and demonstrate capacity for future affinity capture studies. The static uptake of positively-charged FITC-dyed SNPS by HEP–AAO was approximately 4.5× that of NH_2_–AAO (binding/release cycle 1), driven by the negative charge of the sulfate groups of heparin.

Subsequent cycles of binding and release (three cycles) were used to demonstrate reversibility of the electrostatic interactions and accessibility of the pore structure. The highly structured straight-channel pores allowed an estimate of the theoretical maximum uptake of particles based on particle size. HEP–AAO membranes retained reusability across triplicate experiments, utilizing on average of 55% the theoretical capacity of the membranes. Prior reports of the effects of *d*_pore_/*d*_particle_ (>10 in this case) suggest that size exclusion should be negligible for this SNP/AAO combination.^62,63^ The release of captured particles and membrane reuse is consistent with electrostatic particle capture due to interactions with the functionalized pore walls^59,62,64^ and not filtration due to size exclusion.

Bioaffinity capture of a model protein, FGF2, was demonstrated using the immobilized heparin on AAO. Under static adsorption, HEP–AAO captured more protein than AAO (16 : 1), but the capture by NH_2_–AAO suggested nonspecific protein binding (10 : 1). Capping the residual primary amines with a terminal carboxylate group (RCOO–NH_2_–AAO) significantly decreased the adsorption of FGF2 while maintaining high levels of uptake on the heparin-functionalized AAO (RCOO–HEP–AAO). The capture of FGF2 by RCOO–NH_2_–AAO was similar to the background of bare AAO.

Preliminary experiments demonstrate the capture and release of AAV2 from pre-processed cell lysate using HEP–AAO membranes with negligible retention of protein and DNA impurities following a rinse step. Further, the mechanism of this capture and release is not ultrafiltration, as indicated by the ability to effectively retain the capsids during rinsing and release AAV2 using charge screening. Membrane fouling was not observed during the capture, rinse, and release steps in TFF mode. The role of electrostatic interactions on the retention of AAV2 could be further explored by comparing AAV2 capture on HEP–AAO to that of NH_2_–AAO. Nonspecific binding could be reduced through amine capping of the heparinized membrane and the impact on AAV2 recovery explored.

AAV2 capture utilized only a small fraction of interaction sites of the AAO membrane (about 2%, based on steric arguments). The operational conditions for AAV2 capture, the capture, rinse and release steps could be further investigated. Optimization of these conditions is expected to result in increased recovery and final AAV2 titer concentration.^65^ Similarly, the recovery of AAV2 using an affinity capture membrane was presented as the final purification step of clarified AAV2 cell lysate. Integration of the parameters for affinity capture within the membrane-based separation train is an important focus for future studies.^57^ Additional studies of membrane cleaning, regeneration, and stability are required to demonstrate the commercial potential of these functionalized ceramic membranes.

This investigation highlights the use of structured ceramic membranes with straight pore channels for electrostatic and affinity capture of biocolloids for purification of biologics and suggest advantages relative to traditional polymeric membranes and resins.^58^ The loading and release of AAV2 from the nontortuous pores was predictable from pore geometry, from which nanoparticle binding capacity was estimated and the potential for the alternative filtering separation mechanism minimized. The versatile surface functionalization strategies of ceramic supports coupled with reversible electrostatic and affinity binding in an accessible pore structure further highlights the potential of ceramic membranes for applications beyond biologics purification, such as nanoparticle recovery and analysis.^66–70^

## Author contributions

Conceptualization: KED, BLK, LDM, SER. Funding acquisition: MC, RED, BLK, SER. Investigation: KED, MLB, LDM. Methodology: MC, KED, KLE, MLB, LDM. Project administration: MC, BLK, SER, RED. Resources: RED. Supervision: BLK, SER. Writing – original draft: KED. Writing – review and editing: KED, BLK, SER.

## Conflicts of interest

There are no conflicts to declare.

## Supplementary Material

RA-016-D6RA07687D-s001

## Data Availability

Data for this article are available at Open Science Framework at https://doi.org/10.17605/OSF.IO/SP5RU. Files include absorbance values used for quantification of functional groups and raw flux data in .csv text files. Supplementary information (SI): calibration curves for quantification dyes, fluorescently-labelled silica nanoparticles, and fluorescent reporter antibody used to quantify AAO functionalization, visualization of dye uptake and release on functionalized AAO, calculation of theoretical SNP capacity for AAO supports, and calibration curves for the quantification of AAV2, protein, and DNA in clarified cell lysate processed using HEP–AAO. See DOI: https://doi.org/10.1039/d6ra07687d.
