# Supplementary material for: Heparinization of anodized aluminum oxide (AAO) membranes for affinity separations of biocolloids
Source: RSC Adv. 2026 Oct 20;16(48):64837–47. doi: 10.1039/d6ra07687d (PMC13682590; doi:10.1039/d6ra07687d)
Supplement: Supplementary file 1 [file RA-016-D6RA07687D-s001.pdf]

Supplementary Information (SI) for RSC Advances.
This journal is © The Royal Society of Chemistry

Supporting Information

Heparinization of Anodized Aluminum Oxide (AAO) Membranes for Affinity Separations of Biocolloids

Kayla E. Daugherty,[a] Logan D. Martin,[a] Mara Leach-Baughman,[a] Kearstin L. Edmonds,[b] Rebecca E. Dutch,[b] Malgorzata Chwatko,[a] Stephen E. Rankin,*[a] and Barbara L. Knutson*[a]

[a] University of Kentucky, Department of Chemical & Materials Engineering, Lexington, KY, 40406-0046, USA

[b] University of Kentucky, Department of Molecular and Cellular Biochemistry, Lexington, KY, USA

*Corresponding Authors E-mails: stephen.rankin@uky.edu (S.E.R) and bknut2@uky.edu (B.L.K)

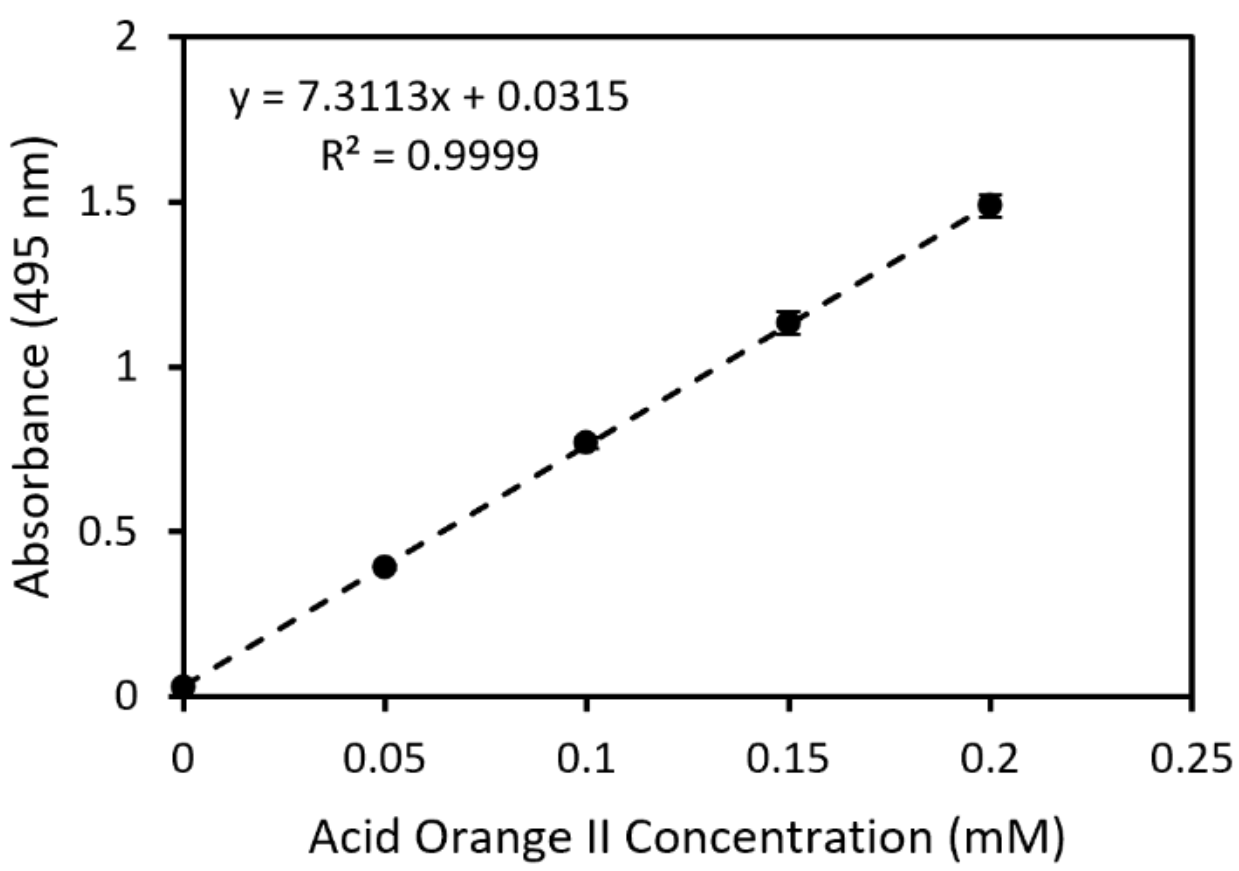

Figure S1. AO II Calibration Curve. AO II concentration in pH 12 H_2O determined via UV-VIS spectroscopy at wavelength 495 nm.

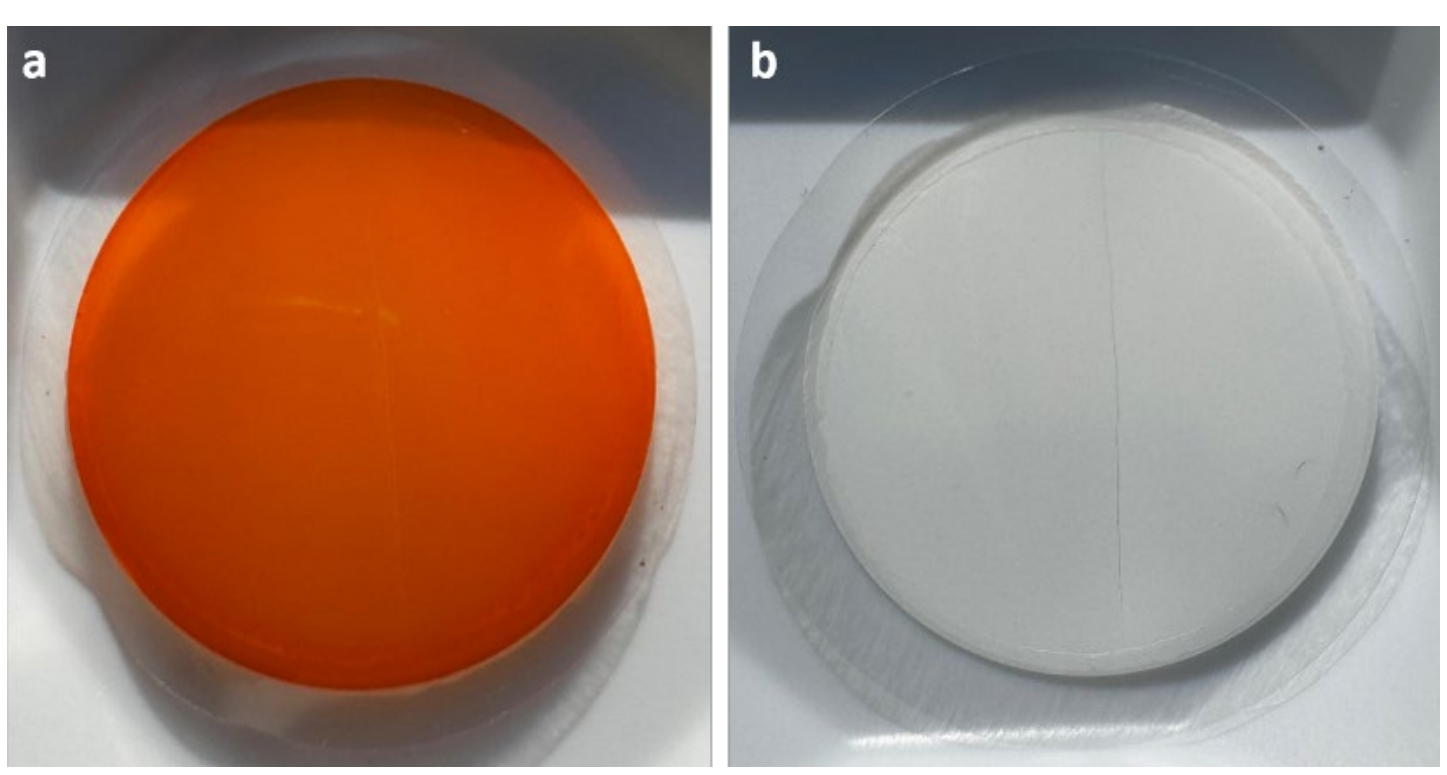

Figure S2. NH_2-AAO during AO II assay. S2a is of a NH_2-AAO after AO II loading. S2b is after dye release at pH 12.

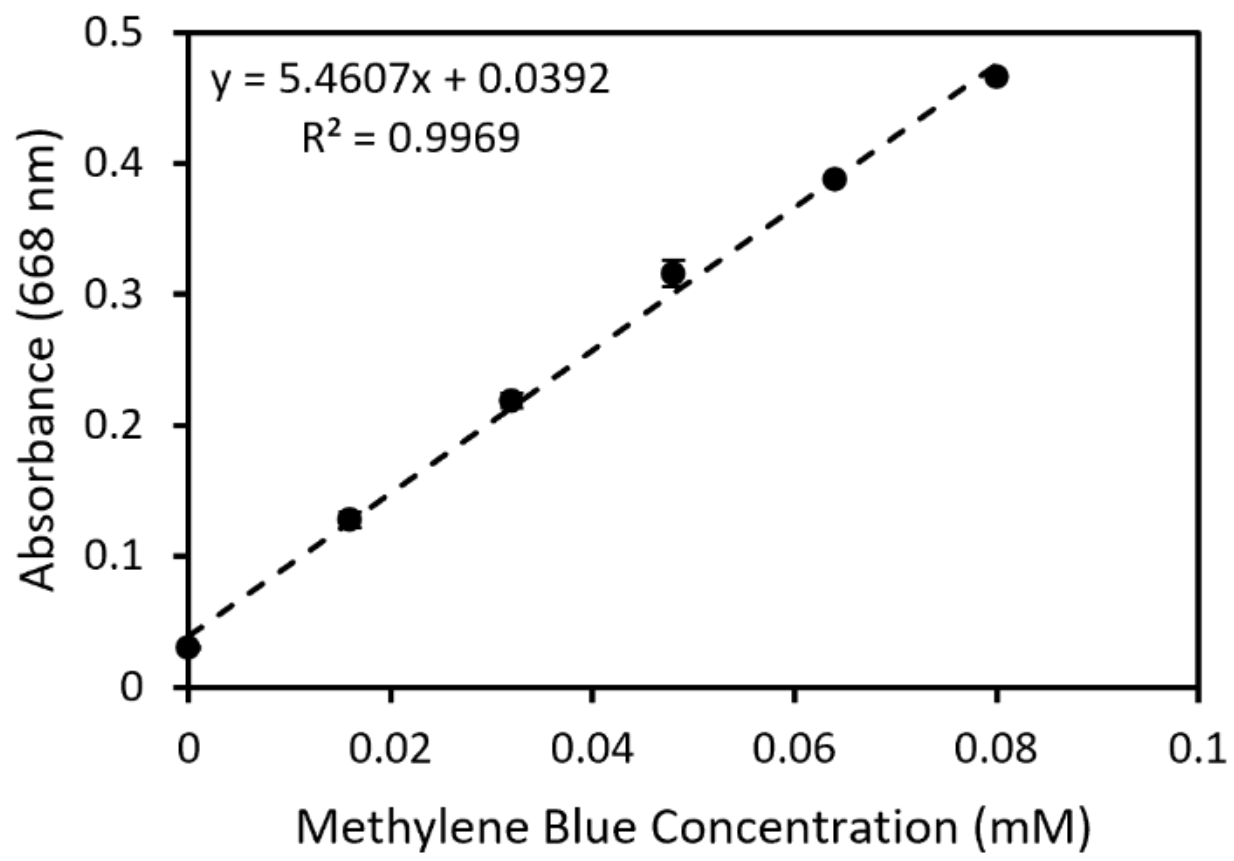

Figure S3. MB Calibration Curve. MB concentration in 0.5M NaCl determined via UV-VIS spectroscopy at wavelength 668 nm.

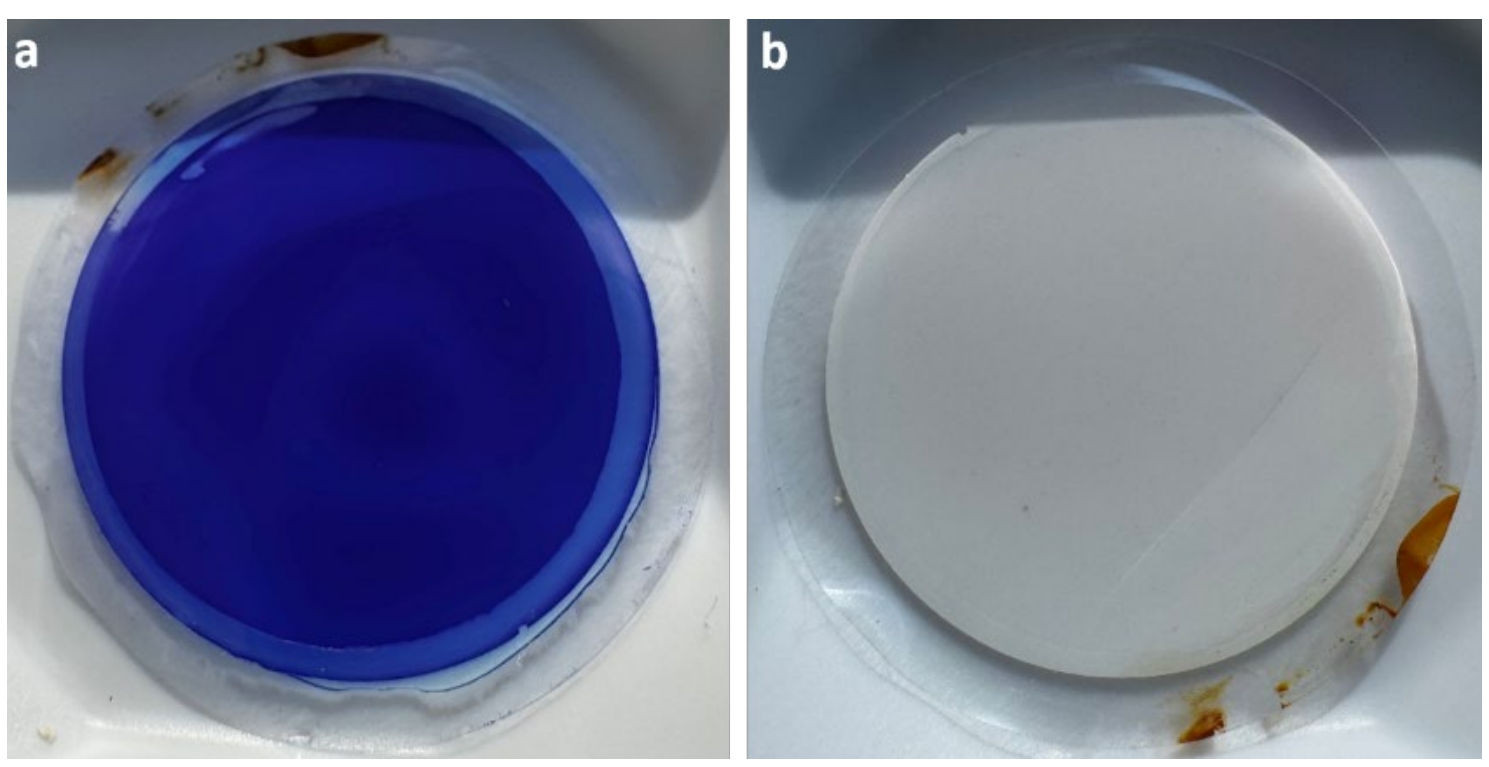

Figure S4. HEP-AAO during MB assay. S4a is of a HEP-AAO after MB loading. S4b is after dye release with 0.5M NaCl.

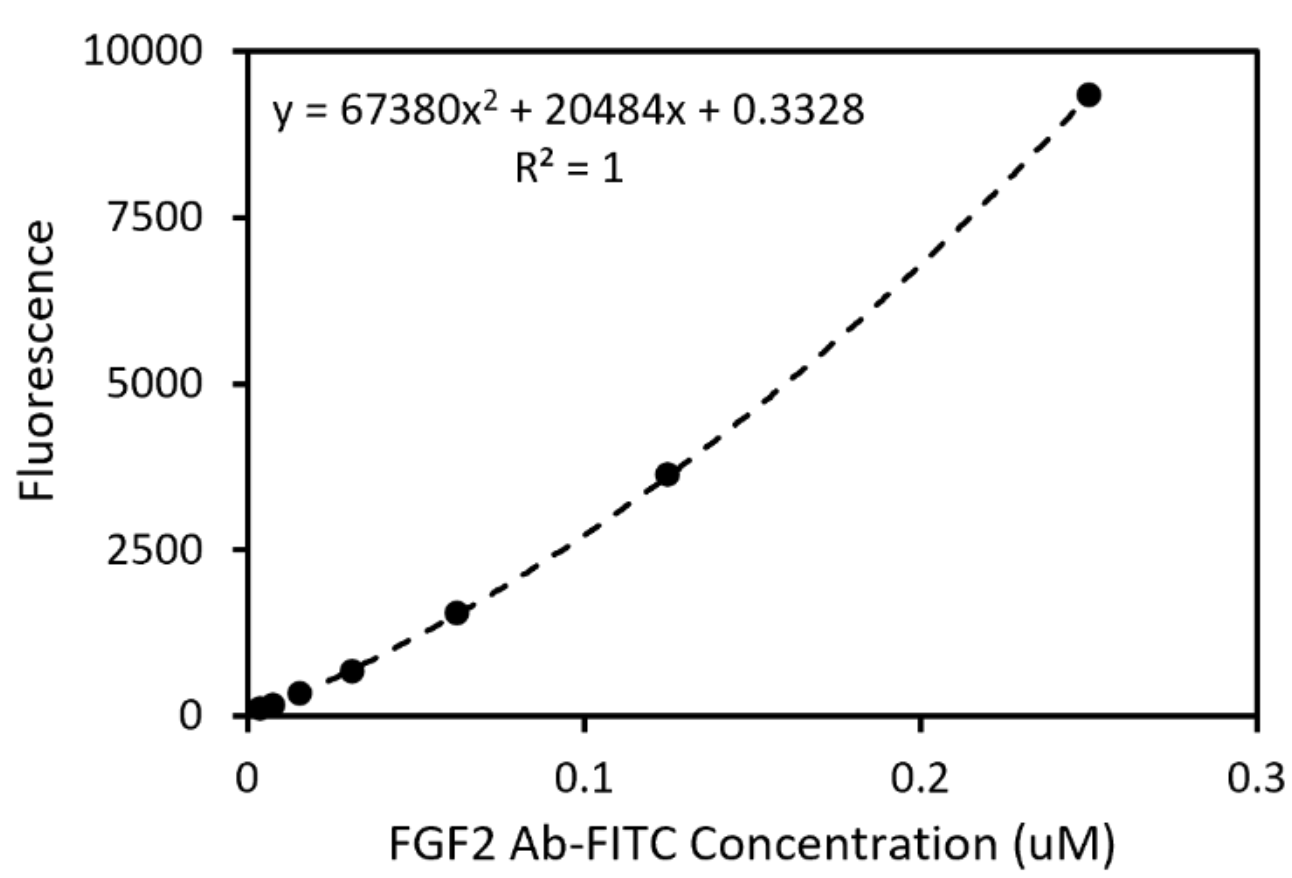

Figure S5. FGF2 Ab-FITC Calibration Curve. FGF2 Ab-FITC concentration in 2M NaCl 50mM Tris-HCl determined with fluorescent spectroscopy at excitation/emission wavelengths 490/520 nm for bioaffinity experiment with AAO, NH_2-AAO, and HEP-AAO.

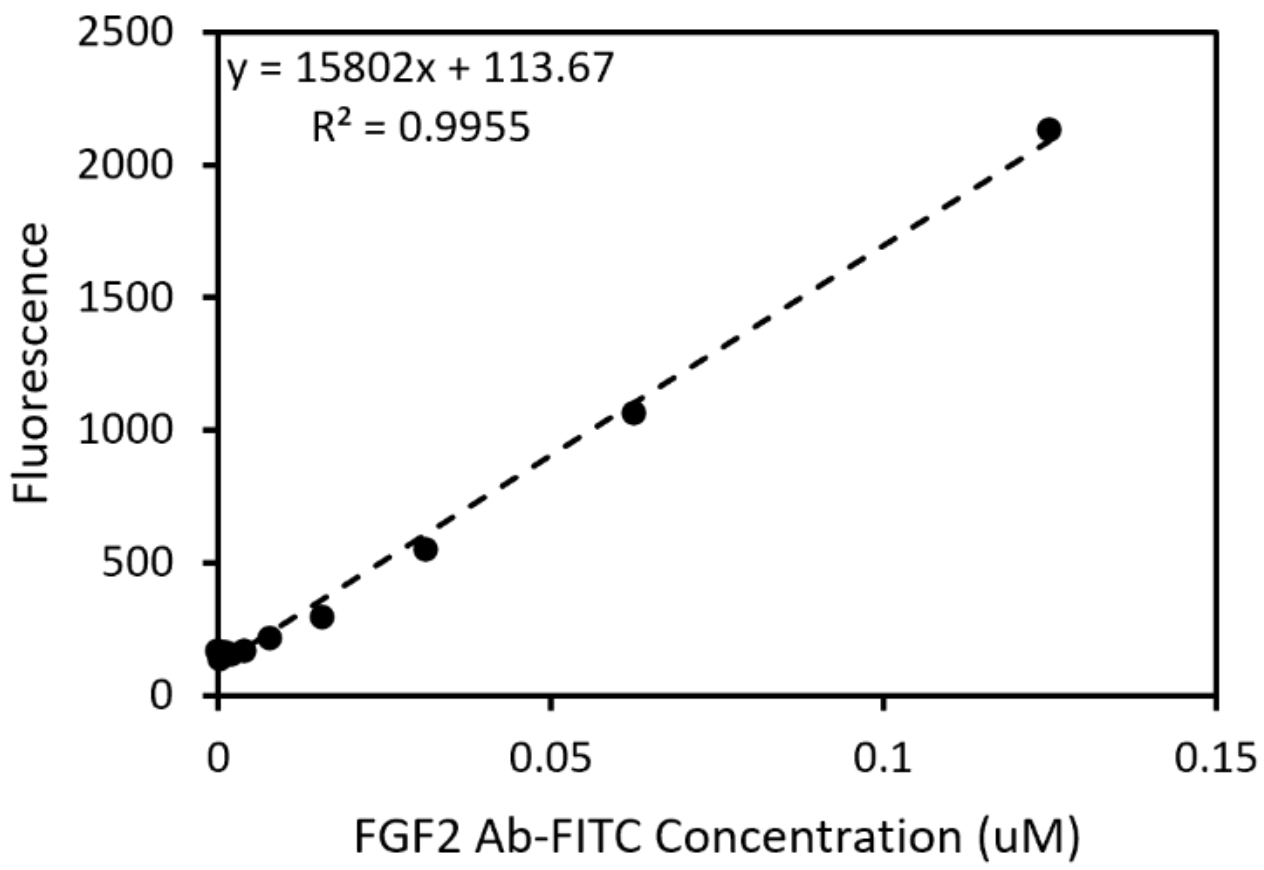

Figure S6. FGF2 Ab-FITC Calibration Curve. FGF2 Ab-FITC concentration in 2M NaCl 50mM Tris-HCl pH 8 determined with fluorescent spectroscopy at excitation/emission wavelengths 490/520 nm for bioaffinity experiment with RCOO-NH_2-AAO and RCOO-HEP-AAO.

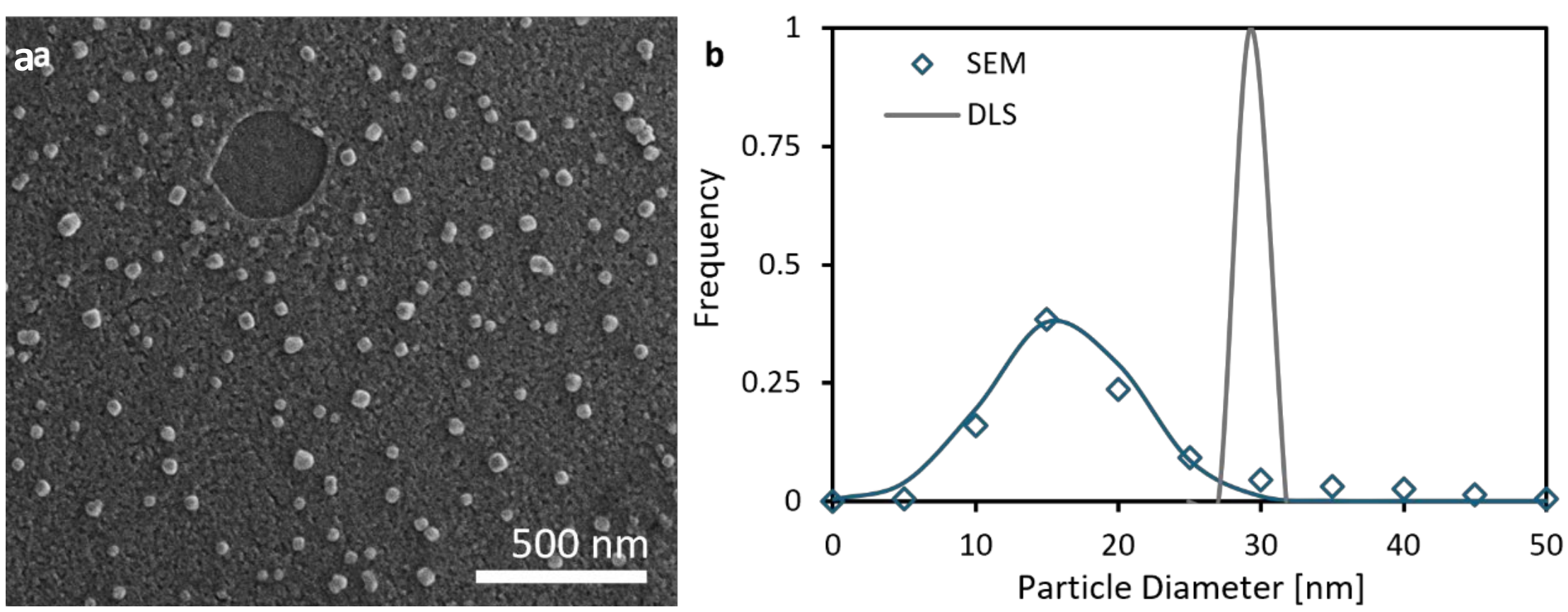

Figure S7. Characterization of SNPs. S7a is a SEM image of synthesized SNPs. S7b are the normalized frequencies of particle diameters obtained with SEM and DLS. SNP diameter from ImageJ measurements of SEM images was 16.1 $\pm$ 5.1 nm (n = 1200). The hydrated diameter measured by DLS was determined to be 29.3 nm.

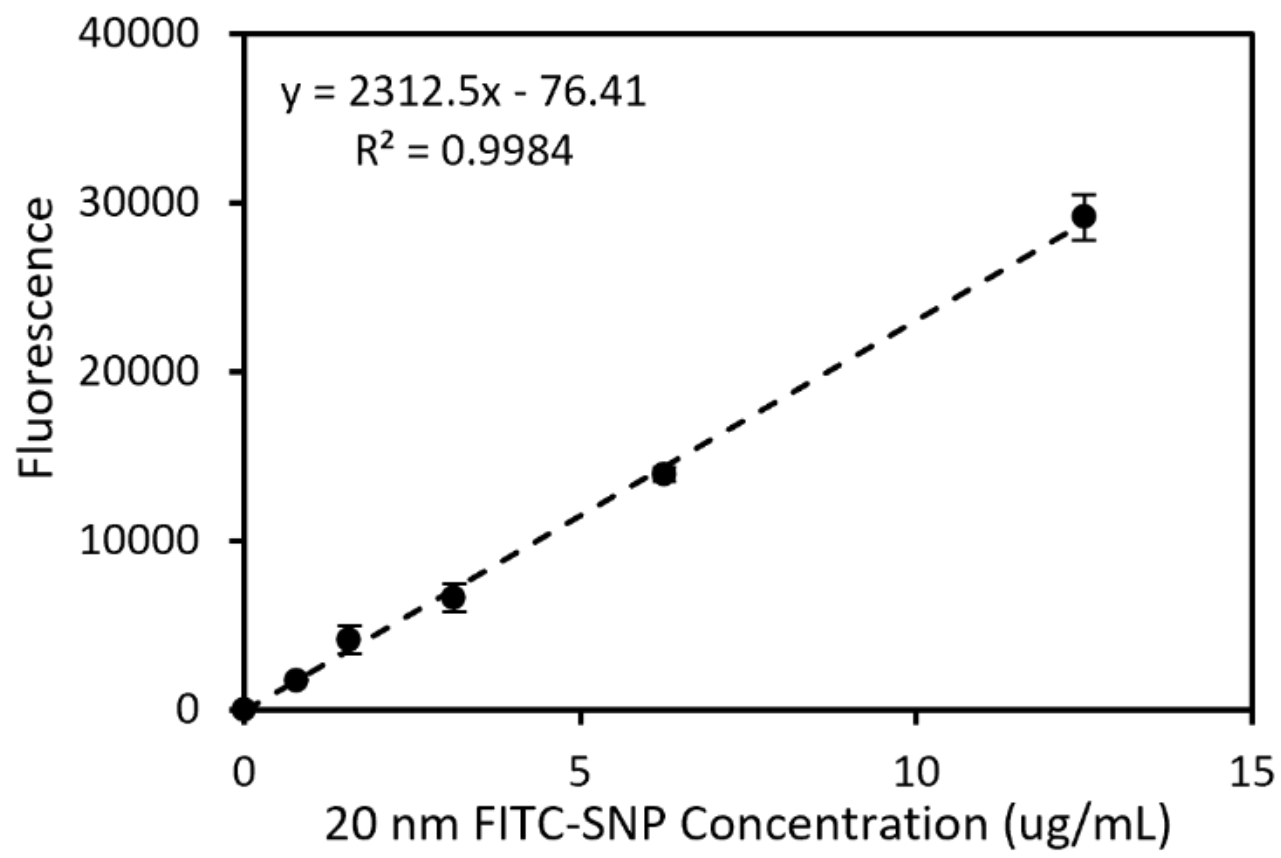

Figure S8. 20 nm FITC-SNPs Calibration Curve. 20nm FITC-SNPs concentration in 1M NaCl determined with fluorescent spectroscopy at excitation/emission wavelengths 490/520 nm.

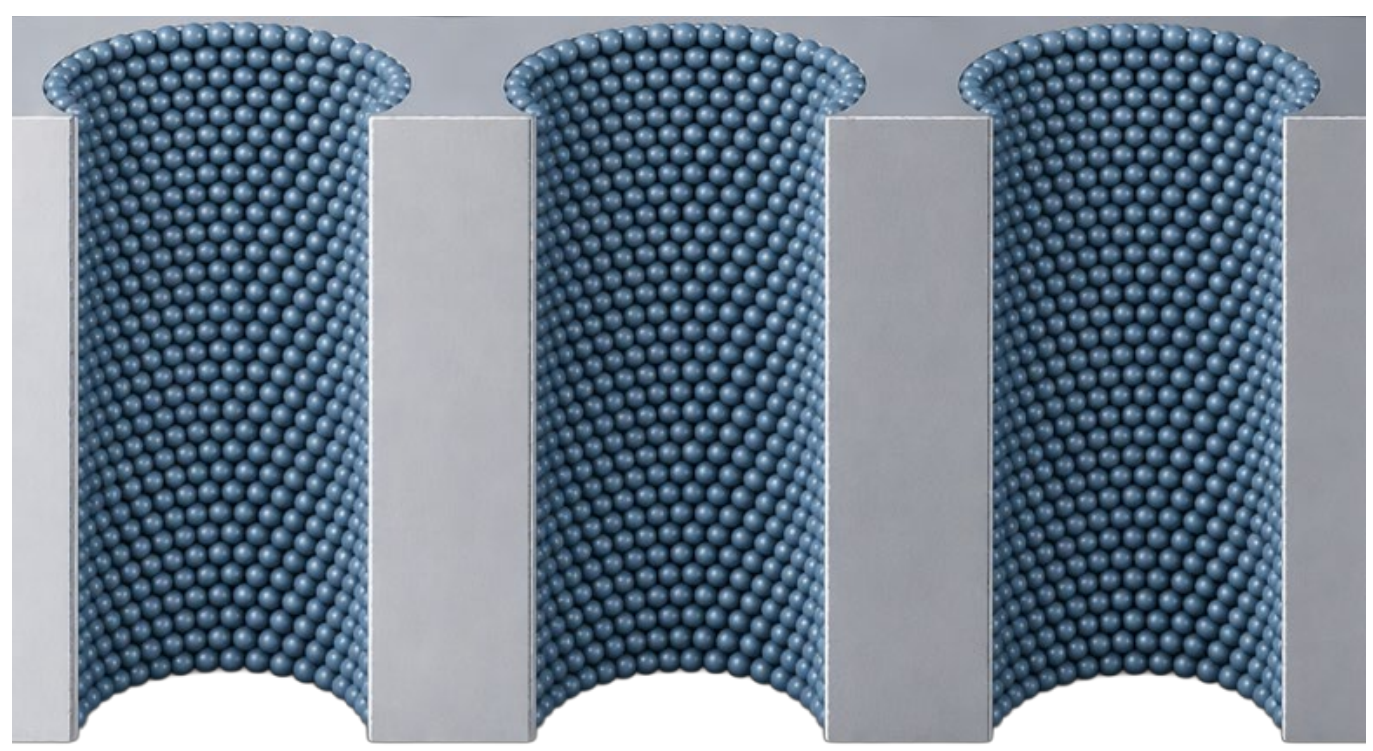

Figure S9. Schematic of a 220 nm pore array lined with close-packed 16 nm spherical particles. This is the geometry used to calculate the maximum capacity of AAO membranes if they capture only a monolayer of SNPA-FITC on the pore wall.

Calculation of maximum particle capacity

Based on the AAO characterization, pore diameter = d_{pore} = 224 nm, pore length = L = 60 μm and porosity = ϕ = 0.63. The particle diameter = d_{SNP} = 16 nm and the assumed silica particle density = ρ = 2 g/cm^3.

The particle cross sections are packed in a pore displaced one particle radius away from the pore wall, which gives an accessible pore diameter of d_{pore}-d_{SNP} = d_{acc} = 208 nm.

For a single pore,

$$A_{pore} = \pi d_{acc} L = 3.92 \times 10^{-11}\ m^2$$

The equatorial area of a particle is :

$$A_{SNP} = \pi (d_{SNP}/2)^2 = 8.94 \times 10^{-16}\ m^2$$

Therefore, the number of particles that a pore can bind, assuming a packing factor of 0.9, is:

$$N_{pore} = 0.9 * \frac{A_{pore}}{A_{SNP}} = 39{,}500$$

Based on the pore cross sectional area (A_{cs}) and the porosity, the pore density per external membrane area is calculated as

$$q_{pore} = \frac{1}{A_{cs}} \cdot \phi = \frac{\phi}{\pi\left(\frac{d_{pore}}{2}\right)^2} = 1.60 \times 10^{13}\ m^{-2}$$

The active diameter of the membrane is 21 mm, so the external membrane area (A_{ex}) is 3.46×10^{-4} m^2. The total particle capacity of a membrane is therefore given by:

$$A_{ex} q_{pore} N_{pore} = 2.19 \times 10^{14}\ particles$$

The mass of one particle is $(4/3)\pi(d_{SNP}/2)^3 * \rho = 4.29 \times 10^{-12}$ μg. Therefore, the total capacity of particles is estimated as (2.19×10^{14} particles)*(4.29×10^{-12} μg/particle) = **938 μg**.

In terms of areal capacities, the internal pore surface area is $A_{ex} q_{pore} A_{pore}$ = 0.217 m^2, so the particles per pore area = 2.19×10^{14} particles/(0.217 m^2) = 1.01×10^{15} particles / (internal pore m^2). For membrane scale up, capacity is expressed per unit of superficial (external) membrane area, or $q_{pore} N_{pore} = 6.31 \times 10^{17}$ particles / (external membrane m^2).

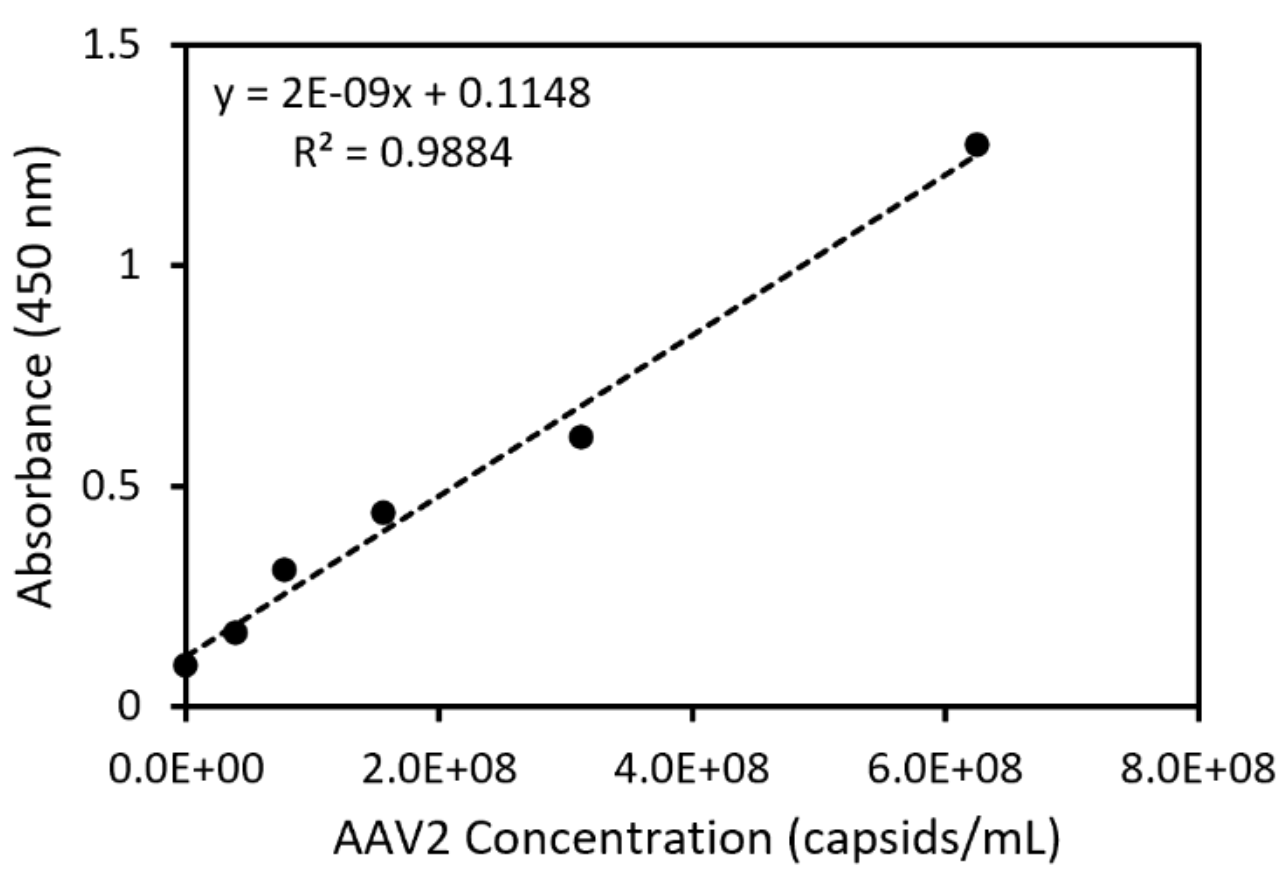

Figure S10. AAV2 Calibration Curve. AAV2 titers in PBS (pH 6) determined via UV-VIS spectroscopy at wavelength 450 nm.

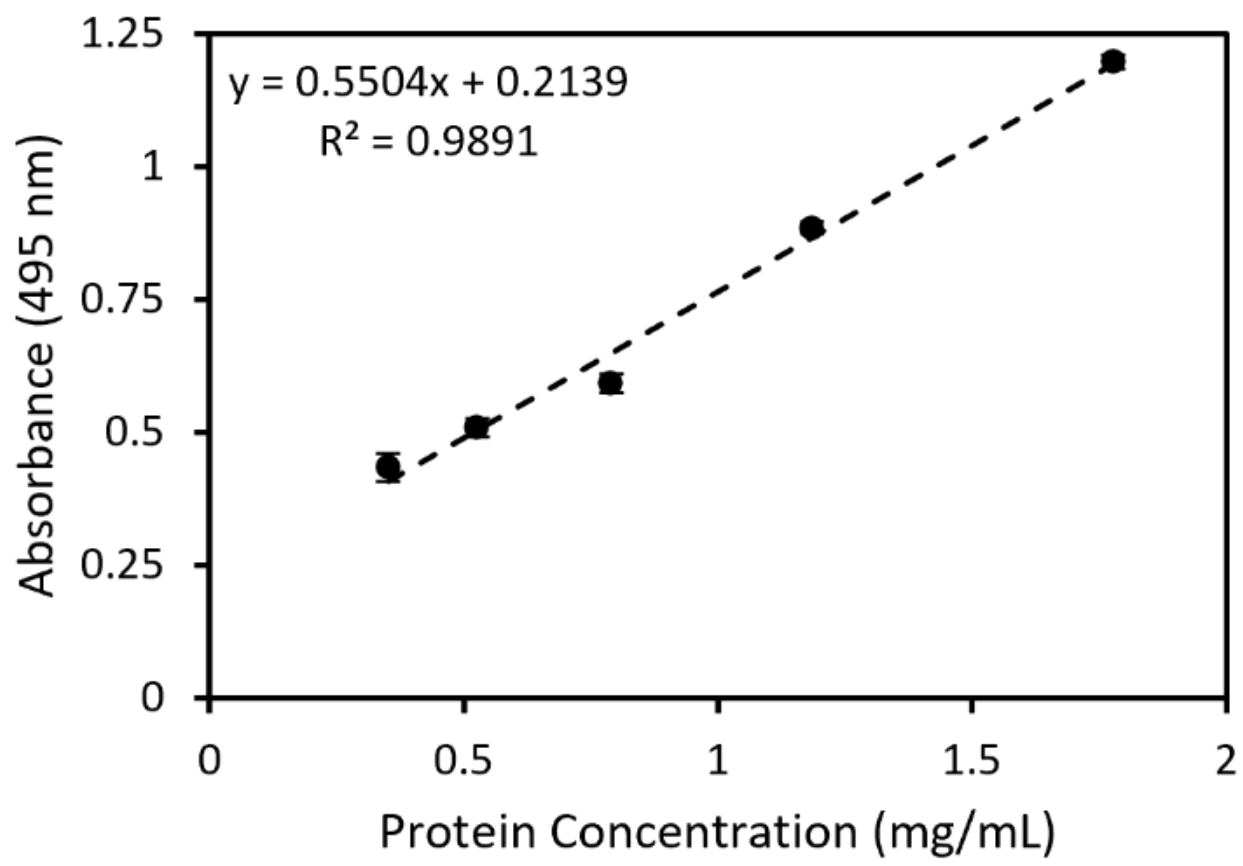

Figure S11. Protein Calibration Curve. BSA concentration in PBS (pH 6) determined via UV-VIS spectroscopy at wavelength 595 nm.

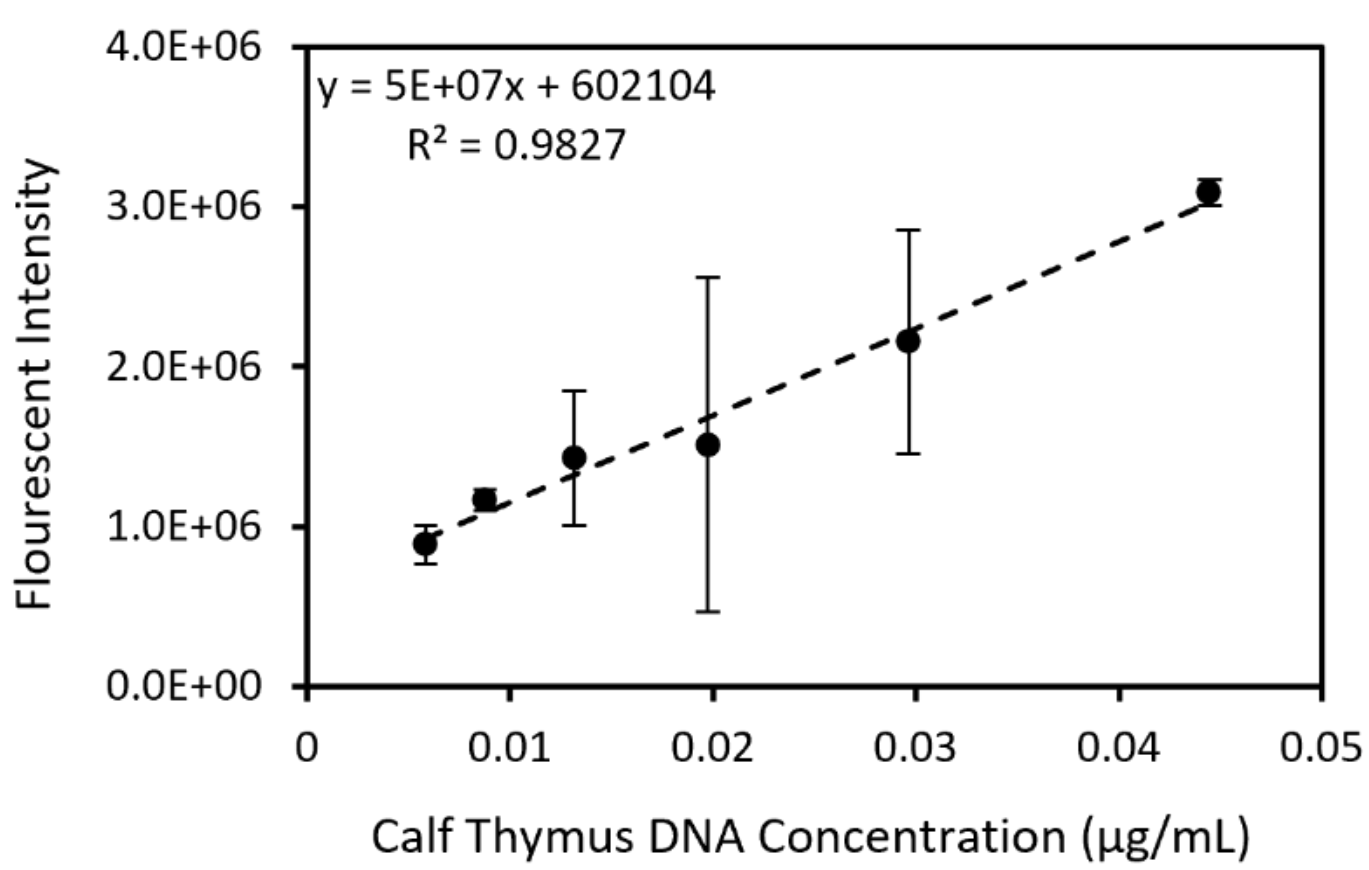

Figure S12. DNA Calibration Curve. Calf thymus DNA concentration in PBS (pH 6) determined through fluorescent spectroscopy at excitation/emission wavelengths 360/460 nm.
